# Therapeutic Approaches to Treat Mitochondrial Diseases: “One-Size-Fits-All” and “Precision Medicine” Strategies

**DOI:** 10.3390/pharmaceutics12111083

**Published:** 2020-11-11

**Authors:** Emanuela Bottani, Costanza Lamperti, Alessandro Prigione, Valeria Tiranti, Nicola Persico, Dario Brunetti

**Affiliations:** 1 Department of Diagnostics and Public Health, Section of Pharmacology, University of Verona, 37134 Verona, Italy; 2 Medical Genetics and Neurogenetics Unit, Fondazione IRCCS Istituto Neurologico C. Besta, 20126 Milan, Italy; costanza.lamperti@istituto-besta.it (C.L.); valeria.tiranti@istituto-besta.it (V.T.); 3 Department of General Pediatrics, Neonatology, and Pediatric Cardiology, University Clinic Düsseldorf (UKD), Heinrich Heine University (HHU), 40225 Dusseldorf, Germany; Alessandro.Prigione@med.uni-duesseldorf.de; 4 Department of Clinical Science and Community Health, University of Milan, 20122 Milan, Italy; nicola.persico@unimi.it; 5 Fetal Medicine and Surgery Service, Fondazione IRCCS Ca’ Granda, Ospedale Maggiore Policlinico, 20122 Milan, Italy; 6 Department of Medical Biotechnology and Translational Medicine, University of Milan, 20129 Milan, Italy

**Keywords:** mitochondria, mitochondrial DNA, mitochondrial disorders, pharmacological therapy, gene therapy, precision medicine

## Abstract

Primary mitochondrial diseases (PMD) refer to a group of severe, often inherited genetic conditions due to mutations in the mitochondrial genome or in the nuclear genes encoding for proteins involved in oxidative phosphorylation (OXPHOS). The mutations hamper the last step of aerobic metabolism, affecting the primary source of cellular ATP synthesis. Mitochondrial diseases are characterized by extremely heterogeneous symptoms, ranging from organ-specific to multisystemic dysfunction with different clinical courses. The limited information of the natural history, the limitations of currently available preclinical models, coupled with the large variability of phenotypical presentations of PMD patients, have strongly penalized the development of effective therapies. However, new therapeutic strategies have been emerging, often with promising preclinical and clinical results. Here we review the state of the art on experimental treatments for mitochondrial diseases, presenting “one-size-fits-all” approaches and precision medicine strategies. Finally, we propose novel perspective therapeutic plans, either based on preclinical studies or currently used for other genetic or metabolic diseases that could be transferred to PMD.

## 1. Genetics of Mitochondrial Diseases

Primary mitochondrial disorders (PMD) are a group of rare diseases affecting approximately 1 in 4300 live birth and causing progressive, incurable defects often resulting in premature death. PMD are characterized by a high genetic, biochemical, and clinical complexity that arise from the dysfunction of the oxidative phosphorylation (OXPHOS), the essential, final pathway for aerobic metabolism [1]. Such impairment is caused by mutations in genes encoding for proteins involved in mitochondrial respiratory chain (MRC) biogenesis, (i.e., subunits of MRC complexes, assembly factors, or post-assembly quality controllers), or by mutations in genes involved in other mitochondrial functions, including fission and fusion machinery, mitochondrial DNA (mtDNA) maintenance, heme biosynthesis, and iron/sulfur metabolism, among others [2].

Each mitochondrion contains several mitochondrial DNA (mtDNA) molecules, the abundance of which spans from hundreds to thousands of copies per cell, depending on the tissue type. For years, it was trusted that in physiological conditions, all mtDNA molecules have the same sequence, a condition known as homoplasmy. When mutations in mtDNA occur, wild type and mutant molecules coexist in the same cell/organ, a condition known as heteroplasmy. However, recent deep resequencing analysis has shown that low-level mtDNA heteroplasmy is extremely common in humans, and, at very low levels (0.5–1%), heteroplasmy seems to be a universal finding [3].

MtDNA mutations could affect any gene encoding the 13 core subunits of the MRC complexes, the 22 mitochondrial tRNAs, or the two rRNAs. They can include point mutations, either homo- or heteroplasmic, and (invariably heteroplasmic) large-scale rearrangements. Heteroplasmic mutations lead to different clinical phenotypes, such as Leigh syndrome (LS) [4], myoclonic epilepsy with ragged red fibers (MERRF) [5], mitochondrial encephalomyopathy with lactic acidosis and stroke-like episodes (MELAS) [6], and neurogenic weakness, ataxia, and retinitis pigmentosa (NARP) [7]. The central disease entity associated with homoplasmic mtDNA mutations is Leber’s hereditary optic neuropathy (LHON) [8]. Mutations in the same gene can lead to different clinical presentation; for instance, mitochondrial phenotypes described in patients with MT-ATP6 mutations span from maternally inherited Leigh syndrome and neurogenic muscle weakness, ataxia, and retinitis pigmentosa (NARP)[9], to Charcot–Marie–Tooth disease [10], late-onset hereditary spastic paraplegia-like disorder [11], and MERRF-like phenotype [12]. Rearrangements (single deletions or duplications) of mtDNA are responsible for sporadic progressive external ophthalmoplegia (PEO), Kearns–Sayre syndrome (KSS) [13], and Pearson’s syndrome [14,15].

There are approximately 1500 predicted mitochondrial genes, that if mutated could lead to mitochondrial dysfunction [16], and so far, more than 300 have been linked to mitochondrial disorders [17,18]. Advanced diagnostic technologies, like next-generation sequencing, are leading to a rapid escalation in the discoveries of novel disease-causing genes. Therefore, a further, widely accepted, genetic classification of PMD is based on the function of the protein products encoded by the mutated genes and includes (i) structural subunits of complexes I-IV, and ATP synthase complex F_0_F_1_; (ii) assembly factors of complexes I-IV, and ATP synthase complex F_0_F_1_; (iii) factors performing or regulating replication, expression, and stability of mtDNA; (iv) proteins related to mitochondrial biogenesis or indirectly associated to OXPHOS; (v) proteins of the execution pathways, such as fission/fusion and apoptosis; (vi) proteins involved in the biosynthesis and metabolism of cofactors; or (vii) proteins involved in the biosynthesis and metabolism of mitochondrial membrane lipids.

The mitochondrial membrane potential (ΔΨm) generated by proton-pumping Complexes I, III, and IV, is essential not only for the energy storage but also for the elimination of disabled mitochondria [19]. Genetic defects affecting these respiratory complexes often lead to dysfunctional ΔΨm with significant consequences to the viability of the cells. However, a comprehensive description of clinical features and genetic causes of PMD is beyond the scope of this manuscript; please refer to [20,21] for an exhaustive list.

**Figure 1:**
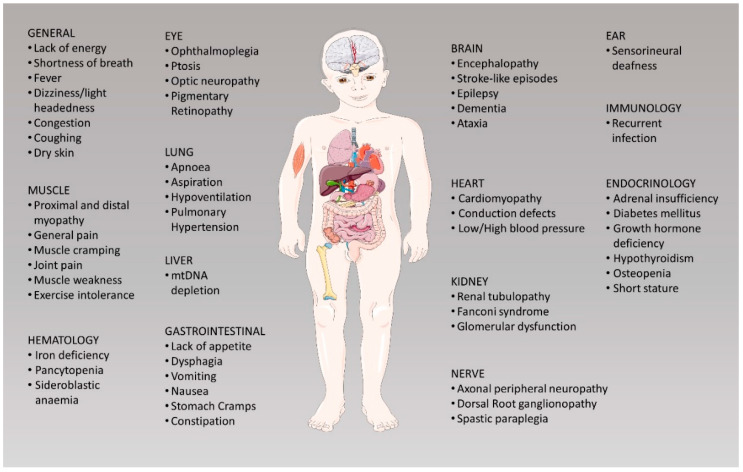
Clinical features of mitochondrial disorder: PMD arise from the dysfunction of the oxidative phosphorylation (OXPHOS) and are characterized by a high genetic, biochemical, and clinical complexity that hinder the prediction of disease progression and the development of therapeutic strategies.

Despite significant advances in understanding the pathophysiology, the extremely varied phenotype–genotype relationship of PMD [1] (Figure 1) has strongly limited the development of effective therapies. Currently, no approved cure exists for most of them, and existing treatments are focused on relieving complications. However, in recent decades, significant signs of progress have been reported. In this manuscript, we will review the most substantial advances in the treatment of PMD and discuss future therapeutic perspectives.

## 2. Therapeutic Approaches to Treat Mitochondrial Disorders

In this manuscript, we choose to classify the current treatments as (i) “one-size-fits-all” strategies, which could, in principle, be used to treat different PMD, regardless the underlying genetic mutation; and (ii) “precision medicine” approaches, aimed at treating a specific PMD with a specific mutation or a peculiar metabolic hallmark. The reader should note, however, that such categorization does not act as unconditional rule, since some exceptions are indeed possible.

The ‘one-size-fits-all’ strategies include symptomatic interventions, mainly diet, exercise, exposure to hypoxia, and pharmacological therapy, which is based on drugs aiming at (i) inducing mitochondrial biogenesis; (ii) stimulating the pathway of nitric oxide synthase; (iii) increasing ATP synthesis; (iv) improving antioxidant defense; (v) enhancing the mitochondrial quality control pathway by stimulating dynamics (fission/fusion events) and degradation of damaged mitochondria (autophagy); (vi) targeting cardiolipin. Recent advances in understanding the underlying pathophysiology of several PMD have made possible the development of a ‘precision medicine’ approach in some cases. Specialized therapies include: (i) supplementation of nucleotides; (ii) replacing defective mtDNA in the oocyte; (iii) supplementation of exogenous mitochondria; (iv) gene- and cell-replacement therapies; (v) scavenging of poisoning metabolites; (vi) organ transplantation; (vii) mtDNA editing. Appendix Table A1 summarizes the most relevant discussed approaches, distinguishing those that are currently only at preclinical level from therapies advanced in clinical trials or exploited in compassionate use.

## 3. “One-Size-Fits-All” Approaches

### 3.1. Physical Exercise

Although mitochondrial patients’ clinical features are highly variable, neurological involvement is often present, best described as neuromuscular defect, including muscle weakness, exercise intolerance, and fatigue. The molecular defect in patients with mitochondrial myopathies (MM) commonly involves the mitochondrial genome, with either single, large-scale deletions, or point mutations, resulting in mosaicism of MRC-competent and MRC-affected muscle fibers [22]. Given the well-established positive effects of physical exercise on healthy subjects, exercise training has been suggested as an approach to improve physical capacity and quality of life. The rationale of endurance training is based on three fundamental aspects: (i) to counteract adverse physiological effects of deconditioning caused by habitual avoidance of activities, provoking the symptoms of fatigue; (ii) to ameliorate the disease process by increasing mitochondrial biogenesis and MRC activity in skeletal muscle; and, in case of mtDNA mutations/deletions; (iii) to stimulate the induction of muscle satellite cells, which have low or undetectable levels of mtDNA mutations, shifting the wild-type mtDNA templates to mature muscle.

Endurance exercise is a potent inducer of mitochondrial biogenesis [23], not only in skeletal muscle but also in the brain [24]. Exercise triggers mitochondrial proliferation through inducing the peroxisome proliferator-activated receptor-γ (PPAR-γ) coactivator 1α (PGC-1α), which is the master transcriptional regulator modulating mitochondrial biogenesis [25]. Endurance training also activates PGC-1β, AMP-dependent kinase (*AMPK*), p38γ MAPK, and hypoxia-inducible factors (HIFs) [26], contributing to the extensive metabolic and molecular remodeling that leads to the preservation of aerobic fitness and muscle strength. Also, physical activity upregulates endothelial nitric oxide synthase (eNOS) gene expression with the consequent increase of nitric oxide (NO) production, which in turn induces mitochondrial biogenesis and cell glucose uptake in skeletal and cardiac muscle [27,28]. Please refer to Section 3.6 for an extensive description of the molecular mechanisms.

Muscle satellite cells are dormant, committed myogenic cells reactivated as needed for muscle growth and repair. Since mutant mtDNA molecules are often undetectable in satellite cells cultured from affected muscles of MM patients [29,30], the stimulations of the proliferation and incorporation of satellite cells into existing myofibers through exercise training have been proposed as a method for normalizing the skeletal muscle mtDNA genotype in MM patients, with encouraging results [31,32]. Favorable effects of physical exercise have been demonstrated in preclinical models of PMD. Prolonged endurance exercise conferred muscular protection and prevented early mortality in the transgenic *PolG* mouse model, harboring a defect in the proofreading-exonuclease activity of mitochondrial polymerase gamma [33]. Significantly, such effects were not limited to skeletal muscle but also involved other organs, including brain, blood, and heart. Exercise training significantly improved aerobic fitness, OXPHOS activity, and muscle strength in the *Harlequin* mutant mouse, a model of complex I (CI) deficiency due to a proviral insertion in the apoptosis-inducing factor (*Aif*) gene. Significant activation of the mTORC1-mediated anabolic pathway in skeletal muscle was reported upon training [34]. Exercise training also remodeled MRC complexes organizations in skeletal muscle of healthy humans, increasing the amount of MRC complexes organized into supercomplexes (SC) and promoting the redistribution of CI from SC I+III_2_ to SC I+III_2_+IV_n_ and of complex III (CIII) and complex IV (CIV) from free forms or SC I+III_2_ into more functional SC species, such as the fully assembled SC I+III_2_+IV_n_ [35].

These and other findings [36,37] supported endurance training as a therapeutic strategy for patients affected by MM, and beneficial effects have been reported in open-label clinical studies. Twelve-week supervised rehabilitation endurance training increased maximal oxygen uptake, work output, minute ventilation, endurance performance, walking distance in shuttle walking test, peripheral muscle strength, and improved clinical symptoms in patients with MM [38]. Prolonged physical exercise increased VO_2MAX_, citrate synthase activity, and mtDNA quantity in muscle biopsies of MM patients; these beneficial effects partially reverted after deconditioning [39]. Taivassalo and co-workers obtained similar training and detraining results, but they did not see an effect on mtDNA amount [40]. Endurance training also promoted heteroplasmic shifting, reducing the relative proportion of mutant to wild-type mtDNA in patients with heteroplasmic mtDNA deletions and point mutations [41,42]. However, other studies reported a trend toward the preferential proliferation of mutant genomes in MM patients with heteroplasmic mtDNA mutations following prolonged aerobic training, despite enhanced muscle OXPHOS, raising some concern proposing endurance training as a treatment option [43]. Finally, other studies suggested that muscle from MM patients may be exposed to greater levels of oxidative stress during the training, given to the reduced expression of DNA repair machinery, and reduced aconitase activity, despite the induction of the antioxidant enzyme Mn-superoxide dismutase (MnSOD) [44].

Therefore, physical exercise could ameliorate clinical conditions and OXPHOS activity in MM patients; however, further studies are needed to investigate whether a link between heteroplasmic shift towards mutant mtDNA and the level of physical activity may exist.

### 3.2. Dietary Approaches

Epilepsy is a common feature of PMD. The ketogenic diet (KD) is a high-fat (≈90%), low-carbohydrate diet that allows the generation of ketone bodies (KB) in the liver through mitochondrial β-oxidation of fatty acids. KB are then metabolized to acetyl-CoA, which feeds the tricarboxylic acid (TCA) cycle, thus serving as an alternative energy source for brain, heart, and skeletal muscle. KD can control seizures with an unclear mechanism [45], and for this reason, it has been proposed for PMD patients who have epilepsy. A comparative study reported that 7 out of 14 children with intractable epilepsy and various MRC complex defects treated with KD became seizure-free, while others had important seizure reduction, spanning 50% and 90% [46]. In another study, KD produced clinical progress, including seizure reduction and global functional improvement in 75% of the treated patients [47]. No severe side effects were reported in both studies.

Besides the curative effects on seizure, KD has also been proposed in patients suffering from inborn errors of pyruvate dehydrogenase complex (PDC) [48], given the alternative production of acetyl-CoA from KB rather than pyruvate. Patients treated with KD showed increased longevity and improved mental development [48]. KD was also proposed to treat CI defects, as it could promote the mitochondrial respiration through complex II (CII) activity and the oxidation of FADH_2_, therefore bypassing the inactive CI [49,50].

KB also increased OXPHOS genes expression through a starvation-like response, resulting in the activation of many transcription factors and cofactors (including AMPK, SIRT1, and PGC-1α), with consequent increase of mitochondrial biogenesis [51]. Additional exciting observations have been reported in cellular models of PMD: KB alleviated mitochondrial dysfunction by restoring CI assembly in cybrid model of MELAS [52], and reduced the mutation load of a heteroplasmic mtDNA deletion in a cybrid cell line from a Kearns–Sayre syndrome patient [53], although the mechanisms of such improvements still need to be deciphered. Similarly, a high-fat diet (HFD) protected fibroblasts with CI deficiency and delayed the neurological phenotype of the *Harlequin* mouse [54]. A preclinical trial in the *Deletor* mouse, overexpressing a mutant replicative helicase Twinkle, revealed slowing of mitochondrial myopathy progression in mice treated with the KD [55]. *BCS1L*-mutated mice with CIII deficiency and progressive hepatopathy fed with KD significantly attenuated liver disease [56]. However, other studies reported that KD could worsen the mitochondrial defect in the *Mpv17* knockout (ko) mouse, characterized by profound mtDNA depletion in the liver [57], in the *Pank2* ko mouse model [58] or in the astrocyte-specific ko mouse of the replicative mtDNA helicase *Twinkle* (*TwKO^astr^*^o^), a model of spongiotic mitochondrial encephalopathy with mtDNA depletion [59].

A modified ketogenic Atkins diet (mAD) was recently tested in patients with MM and progressive external ophthalmoplegia with single or multiple deletions [60]. All patients developed progressive muscle pain and rhabdomyolysis within two weeks; muscle ultrastructure analysis revealed selective fiber damage [60]. These adverse events determined the interruption of the trial. Incredibly, a two-year follow-up showed an increase in muscle strength, suggesting that, following the acute damage, an injury-induced muscle repair by satellite cells—which do not carry deleted mtDNA molecules—was stimulated. [60].

Other dietary approaches include the use of anaplerotic compounds to treat mitochondrial fat oxidation disorders. An example is the odd-chain fatty acid *triheptanoin*, an anaplerotic compound inducing a rapid increase of plasmatic C4- and C5-ketone bodies, the latter being a precursor of propionyl-CoA, which is then converted into succinyl-CoA. Treatment with *triheptanoin* permanently abolished chronic cardiomyopathy, rhabdomyolysis, and muscle weakness in patients with very-long-chain acyl-CoA dehydrogenase (VLCAD) deficiency [61].

In conclusion, in vitro and in vivo shreds of evidence suggest that dietary manipulation promotes different responses in different tissues and cell types, highlighting the need for disease-specific treatments based on their molecular pathophysiology knowledge.

### 3.3. Exposure to Hypoxia

Hypoxia response, a mechanism that helps cells adapting when oxygen is limited, was identified in 2016 as a potent suppressor of mitochondrial dysfunction, and therefore proposed as a therapeutic approach for mitochondrial diseases [62]. The authors identified the inhibition of the Von Hippel-Lindau (VHL) factor as the most effective suppressor of the mitochondrial dysfunction. VHL factor is a critical protein in cellular responses to oxygen availability, being required for the oxygen-dependent proteolysis of α subunits of hypoxia-inducible factor-1 (HIF). VHL factor negatively regulates HIFs, so downregulation of VHL activates the HIF transcriptional response, which induced the partial shift of cellular bioenergetic reliance on mitochondrial OXPHOS [62]. Genetic or pharmacological activation of the HIF pathway in cellular and zebrafish models, as well as chronic normobaric hypoxic treatment in the murine *Ndufs4* ko model of Leigh syndrome, prevented the development of the disease. Hypoxia markedly improved lifespan (from 58 to 270 days), body weight, body temperature, behavior, neuropathology, and disease biomarkers in *Ndufs4* ko mouse, a model of severe infantile Leigh syndrome; on the contrary, hyperoxia (55% O_2_) worsened all the parameters analyzed [62]. Alternate hypoxia/normoxia and moderate hypoxic conditions (17% O_2_) failed to improve the clinical phenotype in *Ndufs4* ko mice [63]. Confirmation of hypoxia’s therapeutic effects in additional models of PMD and an in-depth explanation of the still unclear mechanistic details would open the possibility of pharmacological treatment.

### 3.4. Strategies to Increase ATP Levels

The observation that combined oral administration of febuxostat—an inhibitor of xanthine oxidoreductase (XOR) used to treat gout and hyperuricemia—and inosine elevated both hypoxanthine and ATP levels in peripheral blood of healthy subjects [64], let Kamatani and colleagues hypothesize that PMD patients may potentially benefit from such treatment.

Purine nucleoside phosphorylase catalyzes the conversion of inosine to hypoxanthine, that is further processed to urate by XOR. The concomitant administration of febuxostat and inosine caused a significant increase of the serum hypoxanthine levels. Hypoxanthine is then converted to IMP, while elevated levels of ATP were observed [64,65]. Two PMD patients—one with homoplasmic mutation (m.12192G>A) in the tRNA histidine (*MT*-*TH*) and mitochondrial cardiomyopathy, and the other one with mitochondrial diabetes, carrying a heteroplasmic mutation in tRNA leucine 1 (*MT*-*TL1*)—received concurrent administrations of inosine and febuxostat. In the first case, the specific marker of heart failure brain natriuretic peptide (BNP), was decreased by 31%, and in the second case, the insulinogenic index increased 3.1 times, suggesting a favorable action of the treatment [65]. However, further studies are needed to confirm these findings.

### 3.5. Pharmacological Stimulation of Mitochondrial Biogenesis

Standard features of OXPHOS-related PMD include reduced ATP production and subsequent energy failure. It has been well established that the onset of the clinical phenotype may appear once the residual mitochondrial activity drops below a critical threshold [66]. In this context, the activation of mitochondrial biogenesis could favor the cells residing in affected tissues to improve the ‘mitochondrial energy units’ and ameliorate their energy metabolism. However, debates exist regarding potential harmful consequences of increasing a mitochondrial mass composed mainly of damaged mitochondria, variably characterized by enhanced ROS production and mtDNA damage [67]. Nevertheless, several molecules pharmacologically targeting the mitochondrial biogenesis pathway have been tested in the last decades.

The mitochondrial biogenesis is regulated by a complex signaling cascade requiring coordinated transcription of several proteins encoded by the nuclear and mitochondrial genome. The mitochondrial biogenesis pathway has been extensively investigated in brown adipose tissue and skeletal muscle. The so-called master gene of mitochondrial biogenesis is the Peroxisome proliferator-activated receptor gamma coactivator 1-alpha (*PGC*-*1α*). This transcriptional coactivator physiologically acts as a sensor of various external and internal stimuli (i.e., cold, exercise, nutritional status) to modulate the mitochondrial mass to meet the energy requirements of the cells.

PGC-1α is a transcriptional coactivator of many transcription factors, including the nuclear respiratory factors (NRF1 and NRF2) that control the transcript levels of OXPHOS-related genes [68], the peroxisomal proliferator activator receptors (PPARs) that modulate fatty acid oxidation, as well as estrogen-related receptor α (ERRα) [69], thyroid hormone receptor [70], that modulate thermogenesis but also mitochondrial respiration [71]; transcription factor Yin-Yang 1 (YY1) implicated in respiratory chain expression [72].

Genetic manipulations of preclinical models have confirmed the beneficial effects of the activation of mitochondrial biogenesis pathway in MM. Transgenic mice overexpressing *PGC*-*1α* in skeletal muscle had enhanced endurance performance and a fiber type conversion from type II to type I, with concurrent activation of genes related to mitochondrial oxidative metabolism [73]. When *PGC*-*1α* is overexpressed in the skeletal muscle of *Surf1* ko [74] and other OXPHOS-deficient mice, including *Acta*-*Cox15* ko and *Sco2* knockin-knockout (kiko) mice, a significant amelioration of the phenotypic and molecular aspects of MM occurs (Bottani E, personal observations). On the contrary, selective depletion of *PGC*-*1α* leads to a blunting of exercise-induced increase of MRC proteins in muscle [75,76].

*PGC*-*1α* gene expression is modulated by many stimuli, which share some common molecular pathways despite the proven tissue-specificity of these mechanisms. Some of them include the PKA/CREB pathway, the calmodulin-dependent protein kinase IV (CaMKIV) and calcineurin A (CnA) pathway, and the NOS/cGMP/PGK pathway (for an extensive review, see [25]). Post-translational modifications modulate PGC-1α activity; in particular, it is activated by phosphorylation triggered by AMPK [77] or by deacetylation operated by the nuclear deacetylase Sirtuin 1 (SIRT1) [78]. The pharmacological modulation of both AMPK and SIRT1 activities is possible, and it has been exploited to activate PGC-1α [25]. AMPK is a highly conserved sensor of intracellular adenosine nucleotide levels activated when even modest decreases in ATP production result in relative increases in AMP or ADP. In response, AMPK promotes catabolic pathways to generate more ATP and inhibits anabolic pathways [79]. SIRT1 is an NAD^+^-dependent deacetylase that acts on various substrates and is involved in an extensive assortment of physiological functions, comprising control of gene expression, metabolism, and aging [80].

**Figure 2:**
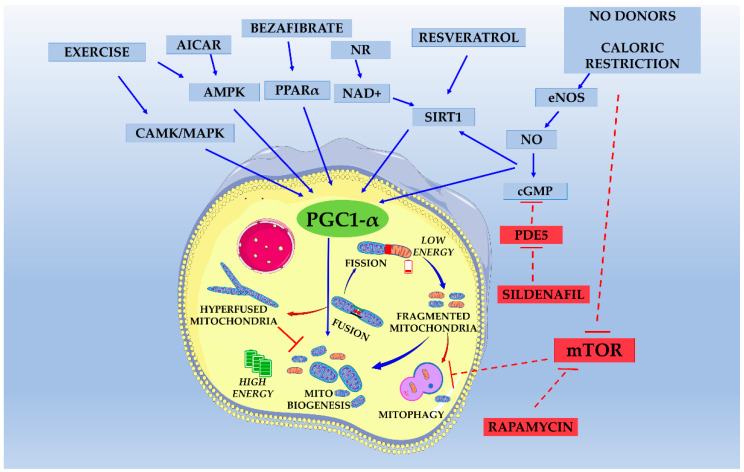
Schematic representation of the pathways regulating mitochondrial biogenesis, dynamics, and mitophagy. External factors (exercise, caloric restriction, or drugs such as bezafibrate, 5-aminoimidazole-4-carboxamide ribonucleoside (AICAR), resveratrol, or nicotinamide riboside (NR) upregulate the expression of *PGC-1α*, which in turn activates essential mitochondrial genes. Other drugs can act on NO pathway (PDE5 inhibitors as Sildenafil) or mitochondrial autophagy (Rapamycin). Blue arrows and squares indicate positive regulations, while red arrows and squares indicate negative regulations.

In the last decade, the PGC-1α signaling cascade has become an attractive therapeutic target to manipulate mitochondrial function, and several drugs acting on the PGC-1α pathway have been tested in preclinical models of PMD [74,81]. A schematic representation of PGC-1α pathway and modulating factors are exemplified in Figure 2.

#### 3.5.1. 5-Aminoimidazole-4-Carboxamide Ribonucleoside (AICAR)

The AMP analogue 5-aminoimidazole-4-carboxamide ribonucleoside (AICAR) has been used to induce PGC-1α-dependent mitochondriogenesis via the activation of the AMPK. We reported a robust induction of OXPHOS-related gene transcription with consequent increase of MRC complex activities in three preclinical models of COX deficiency, a *Surf1* ko mouse, a *Sco2* kiko mouse, and a muscle-specific Cox15 ko mouse [74]. The increase in the MRC activities was paralleled by a significant improvement in the kiko mouse’s motor performance, which has a mild MM, but not in *Acta*-*Cox15* models. This difference is likely due to the severity of the *Acta*-*Cox15* ko mouse model’s clinical phenotype, which could not be corrected despite a clear, although incomplete, rescue of the CIV activity. We also generated an *Acta*-*Cox15* ko mouse model overexpressing PGC-1α in the skeletal muscle. This mouse (*Acta*-*Cox15ko*–*PGC*-*1α*) also showed improved motor performance compared to naive *Acta*-*Cox15* ko littermates, but this effect was transient, and, at six months of age, both *Acta*-*Cox15* ko and *Acta*-*Cox15ko*–*PGC*-*1α* displayed comparable motor performance, suggesting that the overexpression of *PGC-1α* in the skeletal muscle delayed, but did not arrest, the clinical course of the disease. Intriguingly, chronic (three months) AICAR administration improved CIV activity, rescued the motor phenotype, and delayed the onset of the myopathy in a mouse model of slowly progressing MM (*Cox10*-*Mef2c*-*Cre*), either in pre-symptomatic or post-symptomatic administration protocol [82]. However, the authors attributed the effects of AICAR in promoting the regeneration of muscle fibers rather than activation of mitochondrial biogenesis [82]. Interestingly, Golubitzky and co-workers identified AICAR as the most effective compound able to induce mitochondrial biogenesis without altering mitochondrial membrane potential (Δ*ψ*). AICAR also improved growth and ATP content while decreasing ROS production in CI deficient cells [83].

Nevertheless, the use of AICAR to treat CNS disease is limited by its low blood–brain barrier penetrance. Conversely, peripheral stimulation of AMPK and mitochondrial biogenesis may have some beneficial effects on MM.

#### 3.5.2. Bezafibrate and Other PPAR Agonists

Bezafibrate is a fibrate drug, pan-agonist of the isoform alpha of the peroxisome proliferator-activated receptor (PPARα), displaying anti-lipidemic activity. The mechanism of action of fibric acid derivatives was elucidated in the 1990s, after identifying PPARs as targets of these drugs. Upon activation, PPARs bind as obligate heterodimers with the retinoid X receptor (RXR) to specific recognition sequences, called PPAR-response elements (PPRE), in the regulatory region of target genes, leading to cis-activation of gene transcription. There are three isoforms of *PPARs* (α, β/δ, and γ) characterized by different physiological functions and tissue specificity with high expression in liver, heart, and skeletal muscle, where they promote upregulation of genes encoding enzymes of the oxidation pathway [84,85] and fatty acid catabolism [86]. Initial experiments performed by Bastin et al. revealed that bezafibrate administration exerted positive effects on MRC activities. In particular, both control and MRC-deficient patients′ fibroblasts with some residual respiratory chain function level upregulated the expression of several nuclear genes encoding subunits of CI, CIII, or CIV and augmented the enzymatic activities of MRC complexes [87]. These effects were accompanied by the increased expression of PGC-1α, NRF1/2, and Transcription Factor A, Mitochondrial (TFAM), the latter controlling the transcription and replication of the mitochondrial genome. Encouraging results were also obtained in *SCO2* mutant fibroblasts, in which bezafibrate rescued the cytochrome c oxidase (COX) defect [88]. Bezafibrate was also tested in fibroblasts of patients with a de novo heterozygous c.1084G>A (p.G362S) *DNM1L* mutation [89]. In this case, Bezafibrate effectively normalized growth on glucose-free medium, ATP production and oxygen consumption, and improved mitochondrial morphology, although it caused a mild increase in ROS production at the same time [89].

However, controversial preclinical results in the activation of PGC-1α were obtained by using bezafibrate [74,90]. Bezafibrate failed to induce mitochondrial biogenesis in vivo in two different mouse models of COX deficiency [74]. Bezafibrate administration significantly delayed the accumulation of COX-negative fibers and multiple mtDNA deletions in the *Deletor* mouse without inducing mitochondrial biogenesis. On the contrary, mtDNA copy number, transcript, and MRC protein amounts decreased in both *Deletor* and wild-type mice. Furthermore, bezafibrate induced severe lipid oxidation effects, with hepatomegaly and loss of adipose tissue, through a mechanism involving lipid mobilization by high hepatic expression of fibroblast growth factor 21 (FGF21) cytokine [90]. An 8-month bezafibrate treatment of the *Mutator* mouse—a premature aging model that harbors a proofreading-deficient mtDNA polymerase γ—delayed hair loss and improved skin and spleen aging-like phenotypes, without a generalized increase in mitochondrial markers, or improvements in muscle function or lifespan [91].

Recently, the results of an open-label observational experimental medicine study of six patients with MM caused by the m.3243A>G *MTTL1* mutation were published [92]. The aim of this study was to establish preliminary safety and efficacy evidence of bezafibrate on mitochondrial metabolism. No clinically adverse events were reported after the administration of 600–1200 mg bezafibrate daily for 12 weeks to the enrolled patients [92]. A reduction in the number of COX-immunodeficient muscle fibers and improved cardiac function were observed in treated patients. Curiously, some biomarkers, comprising the level of m.3243A>G heteroplasmy in urinary sediments or the exercise physiology, were extremely erratic, explaining why these do not always correlate with clinical severity. Moreover, the known serological biomarkers for PMD, FGF-21, and growth and differentiation factor 15 (GDF-15), were significantly elevated and paralleled by a strong imbalance in amino acid and fatty acid metabolism [92]. This alteration of mitochondrial disease patients’ metabolomic signature following bezafibrate administration suggests being cautious with eventually possible adverse events in long-term treatment.

*Thiazolidinediones* are a class of heterocyclic compounds used to treat type 2 diabetes mellitus that display a high affinity for the PPARγ receptor. Once activated, the PPARγ binds to DNA in conjunction with the RXR receptor, and this heterodimer interacts with transcriptional coactivators, including PGC-1α [93]. Several lines of evidence support the mitochondriogenic effects of thiazolidinediones compounds, both in in vitro and in vivo models [94,95,96]. Interestingly, *Rosiglitazone* (a member of this family) simulated mitochondrial biogenesis in mouse brain through an apolipoprotein (Apo) E isozyme-independent manner. Rosiglitazone induced both mtDNA and estrogen-stimulated related receptor alpha (ESRRA) mRNA, a key regulator of mitochondrial biogenesis. PPARγ agonism induced neuronal mitochondrial biogenesis and glucose utilization, leading to progressed cellular function [97]. However, studies on preclinical models of PMD are lacking.

#### 3.5.3. Modulating NAD^+^ Pool

Another strategy to stimulate mitochondrial biogenesis is based on pharmacological activation of SIRT1, a nuclear deacetylase that utilizes the NAD^+^ moiety to deacetylate acetyl-lysine residues of proteins. Downstream targets of SIRT1 are the Forkhead box O (FOXO), PGC-1α, the myocyte-specific enhancer factor 2 (MEF2), and the tumor suppressor p53 [98], which are involved in the transcriptional regulation of mitochondrial function. As SIRT1 activity is directly regulated by NAD^+^ availability, NAD^+^’s intracellular regulation may represent a strategy to promote SIRT1 activity and its downstream cascade. Various approaches can increase intracellular NAD^+^ concentrations, including (I) supplementation with NAD^+^ precursor [99,100]; (II) pharmacological inhibition of poly (ADP) ribosyl polymerase 1 (Parp1), an NAD^+^ consumer, and SIRT1 competitor [101,102]; or (III) by inhibition of aminocarboxymuconate semialdehyde decarboxylase [103], which results in increased de novo synthesis of NAD^+^ from tryptophan.

Vitamin B3 is a NAD^+^ precursor and exists in several forms: nicotinic acid (niacin), nicotinamide (NAM), and nicotinamide riboside (NR) [100,104,105]. Supplementation with NR and reduction of NAD^+^ consumption by inhibiting the Parp enzymes were tested in *Sco2* kiko mice leading to activation of SIRT1, and induction of OXPHOS genes via the PGC-1α axis, resulting in clinical improvement of the motor performance of treated mice up to normal values [81]. Similarly, NR’s administration activated mitochondrial biogenesis, improved mitochondrial ultrastructure, prevented the generation of multiple mtDNA rearrangements, and delayed the MM in the *Deletor* mouse model [106]. It was also reported that NR administration improved mitochondrial function in iPSC-derived neurons from Parkinson’s Disease (PD) patients and rescued neuronal loss and motor deficits in *GBA-PD Drosophila melanogaster* [107]. Nicotinamide mononucleotide (NMN), another NAD^+^ precursor, significantly extended the lifespan of the *Ndufs4* ko mice by approximately 2-fold [108]. NMN also attenuated NAD^+^ redox imbalance, protein hyperacetylation, and suppressed lactate levels in the skeletal muscle, while brain was not responsive [108].

A recent paper from Katsyuba and co-workers demonstrated that cellular NAD^+^ levels are also controlled by α-amino-β-carboxymuconate-ε-semialdehyde decarboxylase (ACMSD), the enzyme that limits the proportion of ACMS able to undergo spontaneous cyclization in the de novo NAD^+^ synthesis pathway, through a conserved evolutionary mechanism from *C. elegans* to the mouse [103]. RNAi of ACMDS led to increasing mitochondrial mass and respiration, and ultimately lifespan of worms through the activation of the mitochondrial stress response. Moreover, ACMSD inhibitors effectively modulate NAD^+^ levels and mitochondrial function in vitro and in vivo, particularly in mouse models of liver and kidney injury [103].

A systemic NAD^+^ deficiency has recently been reported in patients with an adult-onset type of MM. An elevated dose of niacin (to 750–1000 mg/day) was administered in 416 patients and their matched controls, for 10 or 4 months, respectively (ClinicalTrials.gov identifier NCT03973203). Blood NAD^+^ grew to 8-fold in all participants, and the patients’ muscular NAD^+^ achieved the control level. Mitochondrial mass and muscle strength increased in all patients; furthermore, muscle metabolism normalized, and liver fat dropped by as much as 50% in patients. Lessened concentration of hemoglobin and erythrocytes and increased muscle glycogen have been identified as potential adverse reactions which need focus and follow-up. Similar increases in circulating NAD^+^ were reported with NR treatment in healthy subjects [109]. These data point to a possible interference of NAD^+^ precursors with erythropoiesis and/or iron metabolism, which require an appropriate supervising in the context of B3 supplementation [105]. However, these data suggest niacin as a good candidate to treat MM with NAD^+^ deficiency, whereas possible curative effects on other PMD are still unclear.

#### 3.5.4. I-BET 525762A

Through a high-throughput chemical screen, Barrow and co-workers identified I-BET 525762A, a bromodomain inhibitor, as a top hit that augments COX5a protein levels in CI-mutant cybrid cells. In parallel, bromodomain-containing protein 4 (BRD4), a target of I-BET 525762A, was identified using a genome-wide CRISPR screening to search for genes whose loss of function rescues the death of CI-impaired cybrids. Furthermore, I-BET525762A administration, or loss-of-Brd4, remodeled the mitochondrial proteome and increased the levels and activity of OXPHOS protein complexes, rescuing the CI defects and cell death [110]. BRD4 is a chromatin-bound transcriptional regulator linked to the expression of genes associated with different biological processes, including tumor progression or inflammation [111]. These findings suggest that these programs integrate with mitochondrial energetics and metabolic control, although the precise mechanism(s) needs still to be deciphered.

#### 3.5.5. Polyphenols and Other Pharmacognostic Products

*Resveratrol* (2,3,4′-trihydroxystilbene) is a polyphenol that received attention for a range of potentially beneficial effects, including mitochondrial function improvement, anti-inflammatory properties, and protection against metabolic diseases and neuronal dysfunction. In 2003, resveratrol (RSV) was identified as the most potent SIRT1 activator molecule in a drug screening [112]. Since then, many studies have pointed to the ability of RSV to upregulate the Sirt1-mediated mitochondrial biogenesis and the functions of other key players as AMPK and PGC-1α. Although the effects of RSV in inducing mitochondrial biogenesis are well established and reproducible [113,114], concerns exist on the molecular mechanism(s) of action. Some authors reported that RSV acts primarily on AMPK activation [115,116], and that the activation of SIRT1 by RSV is an artifact [117,118,119]. In line with this hypothesis, Jee-Hyun Um et al. showed that RSV was unable to exert its pharmacological effects in AMPK deficient mice, proving that AMPK is a crucial target of RSV [120]. Other authors instead reported that RSV might first activate SIRT1 in vivo, leading to AMPK activation [121,122], and deacetylation of PGC-1α [114]. However, different RSV dosages preferentially activate SIRT1 or AMPK in vivo, further increasing the complexity of these pathways [113]. Besides the molecular mechanisms, studies based on preclinical neurodegenerative disease models reported positive effects of RSV in improving mitochondrial function and neurological symptoms [123,124]. More recently, the effects of RSV administration on mitochondrial respiration of skin fibroblasts from PMD-patients have been reviewed [125]. It appeared clear that responses to RSV are not uniform but highly patient- or mutation-dependent. Further studies on proper cellular models are needed to evaluate the effects of RSV on PMD.

*Quercetin* is a potent antioxidant flavonoid, more specifically a flavonol, with a reported ability to activate SIRT1 and PGC-1α and increases mtDNA and cytochrome c content in skeletal muscle and brain [126,127]. The use of quercetin for the treatment of neurodegenerative disorders with mitochondrial involvement has been exploited in preclinical models of Alzheimer’s Disease [128] and Parkinson’s disease [129].

*Hydroxytyrosol* (HT) is a polyphenol which activated PGC-1α through SIRT1 de-acetylation and induced mitochondrial biogenesis in vitro [130] and in skeletal muscle in vivo [131]. Furthermore, HT exerted dose-dependent effects on SC assembly in exercised animals through enhancing mitochondrial function [132]. Prolonged HT administration significantly activated AMPK, SIRT1, and PGC-1α, and increased the MRC complexes’ levels in the brain of db/db mice. Likewise, targets of the antioxidative transcription factor nuclear factor erythroid 2 related factor 2 (NRF2), including p62 (sequestosome-1), heme oxygenase 1 (HO-1), and superoxide dismutase 1 and 2 increased, and protein oxidation significantly decreased upon treatment [133]. Recently, by using three different *C. elegans* models of PD, it was shown that HT enhanced locomotion in worms suffering from α-synuclein-expression in muscles or rotenone exposure, reduced α-synuclein accumulation in muscle cells, and prevent neurodegeneration in α-synuclein-containing dopaminergic neurons [134].

*Curcumin*—a dietary polyphenol derived from turmeric—also stimulated different mitochondrial biogenesis markers in vivo when administered to the senescence-accelerated mouse-prone 8 (SAMP8) strain. In particular, curcumin upregulated PGC-1α protein expression in the brain, improving MMP and ATP levels and restoring mitochondrial fusion [135]. In another study, curcumin dietary supplementation increased the expression of TFAM and PGC-1α, and ATP levels in mouse brains [136]. Curcumin also showed antioxidant effects in patients affected by β-Thalassemia [137,138]. Therefore, curcumin was proposed to treat LHON patients in phase 3 clinical trial (ClinicalTrials.gov identifier NCT00528151). Seventy patients with 11,778 LHON mutation were randomly treated with oral curcumin (500 mg/day) or placebo for one year. The visual acuity, computerized visual field, electrophysiologic parameters, and oxidative stress enzymes in plasma were compared before and after treatment at 3-, 6-, and 12-month intervals. Although the study was completed in 2007, results have not been published to date.

Despite the wide choice of molecules with a potential effect on mitochondrial biogenesis, more effort is needed to clarify which drug is most effective in patients affected by PMD. Bezafibrate gave highly variable results; AICAR presents several limitations for chronic use [139,140]; furthermore, the potential mutagenic effects of PARP inhibitors are still to be thoroughly evaluated, although data collected in patients treated with Olaparib (AZD-2281) suggest low mutational toxicity [141]. On the contrary, the possibility of translating into clinical practice supplementation with NAD^+^ precursors seems to be more realistic, based on the high tolerability and substantial lack of adverse effects [142,143,144].

### 3.6. Pharmacological Modulation of the NO/cGMP/PKG Pathway

Nitric oxide (NO) is an intra- and extra-cellular gaseous second messenger that acts on various signaling pathways in target cells and orchestrates a plethora of physiological processes, including neuronal signaling, modulation of ion channels, immune response, inflammation, and cardiovascular homeostasis, among others. NO is catalytically produced from L-arginine and L-citrulline—the latter is converted to L-arginine via argininosuccinate synthase and argininosuccinate lyase—by the three isoforms of the enzyme nitric oxide synthase (NOS): the neuronal NOS (nNOS, or NOS1), inducible NOS (iNOS or NOS2), and endothelial NOS (eNOS or NOS3). Since mitochondria also produce NO, the existence of a putative mitochondrial NOS (mtNOS) is feasible, yet still controversial [145,146,147]. The enzymatic reaction generating NO involves the transfer of electrons from NADPH, via the flavins in the C-terminal reductase domain, to the heme in the N-terminal oxidase domain of NOS, where the substrate L-arginine is oxidized to L-citrulline and NO [148]. Stimulation of NOS leads to the generation and release of NO, which causes the activation of soluble guanylate cyclase (sGC) and cGMP production. The biological effects of cGMP are mediated by three major groups of cellular targets: cGMP-dependent protein kinases (PKGs), cGMP-gated ion channels, and phosphodiesterases (PDEs) [149]. Once activated by cGMP, PKGs initiates a cascade of phosphorylation events on various target proteins, resulting in modification of physiological processes, including calcium homeostasis, smooth muscle contraction, and cardiac function [149]. cGMP-gated channels are non-selective ion channels that function in response to cGMP binding and have important signal transduction roles in retinal photoreceptors and olfactory receptor neurons [150]. However, it should be noted that, other than cGMP-gated channels, many other ion channels are indirectly regulated by cGMP through PKG-consensus motifs on their sequence [151,152]. Lastly, the cGMP level is determined by the balance between sGC and PDEs activities, the latter breaking down cGMP molecules [149]. Notably, it has been reported that the NOS/NO/sGC/cGMP signaling upregulates PGC-1α [153] in diverse cell types, including neurons [154,155]. The mechanisms by which cGMP activate PGC-1α may involve the PKG-driven modulation of the CREB signaling pathway, which has been recently reviewed elsewhere [156].

NO deficiency occurs in PMD and may be due to multiple factors, although not fully elucidated. The first hypothesis points to a generalized impairment of endothelial function, as observed in PMD [157]. Flow-mediated vasodilation (FMD), which is a function of NO synthesized by endothelial cells in response to reperfusion, is impaired in individuals with MM, MELAS, MERRF, MIDD (maternally inherited diabetes and deafness), and CPEO [158]. As often reported, abnormal mitochondrial proliferation may cause NO sequestration by CI and CIV binding [159]. Finally, reduced levels of NO precursors [160,161,162] and of sarcoplasmic NOS activity in COX-negative fibers from patients with PEO, MM, and MELAS syndrome were reported [163]. Beyond the cause(s), NO depletion may play a significant role in the onset of several observed complications, including stroke-like episodes, myopathy, diabetes, and lactic acidosis [164]. Subjects with MELAS syndrome have lower concentrations of NO metabolites (nitrite and nitrate) during stroke-like episodes [165] and low L-citrulline levels, suggesting that MELAS strokes may be caused by unstable NO homeostasis that leads to vascular endothelial dysfunction [161].

#### 3.6.1. L-Arginine and L-Citrulline

As L-arginine and L-citrulline are NO precursors, their supplementation was proposed to treat NO deficiency-related manifestations of PMD [161,166,167]. L-Arginine supplementation increased the NO production rate [164] and improved FMD in MELAS patients [162]. An open-label trial showed that intravenous L-arginine administration to MELAS patients during stroke-like episodes led to an improvement in the clinical symptoms associated with these episodes, and oral L-arginine supplementation at the interictal phase decreased their frequency and severity [165]. A series of open-label studies confirmed these findings in MELAS patients with the common m.3243A>G mutation [162,168]. Interestingly, the NO synthesis rate effectively increased upon L-citrulline supplementation, rather than L-arginine, indicating that L-citrulline is a more powerful NO precursor than L-arginine [164,169]. Moreover, L-arginine and L-citrulline administration reduced plasmatic alanine and lactate concentrations, suggesting that such supplementation may improve lactic acidemia in MELAS syndrome by improving NO-mediated perfusion and oxygen delivery in all microvasculature compartment [167]. So, the L-citrulline and L-arginine supplementation may also be extended to treat other clinical features of PMD, e.g., lactic acidosis, muscle weakness, exercise intolerance, and diabetes. As such, a randomized crossover study (ClinicalTrials.gov identifier NCT02809170 was performed to evaluate the impact of L-citrulline and L-arginine supplementation on endothelial dysfunction in pediatric PMD patients. The primary outcomes were the changes in reactive hyperemic index, which reflects endothelial function, but results are not yet available. Currently, a Phase-1 clinical trial is recruiting patients to establish dose and safety of L-citrulline treatment of NO deficiency in MELAS (ClinicalTrials.gov identifier NCT03952234). Placebo-controlled randomized clinical trials are necessary before L-arginine and L-citrulline can be definitively recommended to ameliorate or treat stroke-like episodes in MELAS and other PMD.

#### 3.6.2. Natriuretic Peptides and Cyclic Guanosine Monophosphate

Natriuretic peptides (NPs) induce natriuresis (i.e., the excretion of sodium by the kidney). NPs regulate vascular tone via GC, cGMP, and PKG [170]. The polypeptide hormones Atrial natriuretic peptide (ANP) and brain natriuretic peptide (BNP) regulate the vascular tone and natriuresis. ANP and BNP stimulate the production of cGMP via a selective binding to their receptors, the natriuretic peptide receptors A and B (NPRA/GC-A and NPRB/GC-B, respectively) which so activate their intracellular guanylate cyclase domains [170]. Transgenic mice overexpressing BNP or PKG increased the mitochondrial muscle content and fat oxidation through upregulation of PGC-1α and PPARδ, preventing obesity and glucose intolerance; moreover, treatment of myotubes with ANP and BNP stimulates mitochondrial biogenesis and mitochondrial respiration [171]. Exercise induced expression of NPRA/GC-A and was correlated with the expression of PGC-1α-dependent genes in muscle [172]. Whitaker et al. showed that phosphodiesterase type-3 (PDE3) inhibitors cilostamide and trequinsin increased PGC-1*α* levels, mRNA expression of mitochondrial genes, and mtDNA copy number both in renal proximal tubular cells and in the renal cortex [173]. However, these compounds have not been tested on PMD models yet, and therefore, future studies are necessary to exploit their potential therapeutic effects.

#### 3.6.3. PDE5 Inhibitors

Sildenafil is the first specific phosphodiesterase type-5 (PDE5) inhibitor (PDE5i) marketed to treat erectile dysfunction. PDE5 is expressed in many tissues where it hydrolyzes intracellular cGMP; thus, PDE5i potentiates the endogenous increase of cGMP by inhibiting its breakdown [174]. Sildenafil restored mitochondrial biogenesis and favored renal recovery in mice after folic acid-induced acute kidney injury [173]. A recent study showed that sildenafil treatment induced mitochondrial biogenesis, increased UCP-1 expression, and promoted subcutaneous white adipose tissue browning in healthy mice [175]. Moreover, PDE5is have emerged from drug screening on MILS-neuronal progenitor cells (NPCs) as the most effective drug to ameliorate mitochondrial function. NPCs derived from patients carrying a deleterious homoplasmic mutation (m.9185T>C) in the mitochondrial gene *MT*-*ATP6* showed defective ATP production and abnormally high mitochondrial membrane potential (MMP), with altered calcium homeostasis [176].

Avanafil, a PDE5i, rescued the calcium defect in patient NPCs and differentiated neurons [176]. However, the NO pathway was not evaluated in this study; instead, a possible link with the activation of Ca^2+^-activated potassium channels mediated by cGMP was speculated [176]. Nevertheless, the beneficial effects of PDE5i to treat PMD are still poorly understood, since preclinical studies [177,178] and case reports [179] gave controversial results; further investigations on the mechanisms need to be implemented.

### 3.7. Antioxidants

Reactive oxygen species (ROS) are unstable molecules containing oxygen that can quickly react with other molecules within cells. They are generated primarily as by-products of the enzymatic activities of the mitochondrial electron transport chain. ROS molecules comprise superoxide, hydrogen peroxide, hydroxyl radical, and hydroxyl ion. Hydrogen peroxide is not as reactive as the hydroxyl radical, yet the latter is readily generated by the former in the presence of Fe^3+^, through the so-called Fenton reaction. Mitochondria are the primary site of ROS production within the cell. In physiological conditions, ROS act as signaling molecules through a tightly regulated process in cell proliferation [180], development, immunity, apoptosis, among others [181], while are scavenged by different antioxidant enzymes that include various isoforms of glutathione peroxidase (GP), superoxide dismutase (SOD), and peroxiredoxin (Prx) [182]. In pathological conditions due to mutations in genes involved in the OXPHOS system, the inefficient transfer of electrons among the four respiratory chain complexes causes an accumulation of electrons that react with molecular oxygen to form superoxide anions (O_2_^−^) [183], superoxide dismutase enzymes then convert that to H_2_O_2_, which can be further reduced to hydroxyl radical (OH^−^), the most potent oxidizing agent among the ROS [183]. Therefore, ROS generation is enhanced, leading to ROS-mediated, irreversible cellular damage, including lipid peroxidation, DNA modifications, and cell death [182]. Moreover, ROS further damage MRC complexes, including NADH dehydrogenase, cytochrome c oxidase, and ATP synthase, and alter mitochondrial membrane permeability and structure, resulting in a complete shutdown of mitochondrial energy production (for a detailed review, see Guo et al. [184]).

Therefore, using antioxidant drugs in mitochondrial disease treatment is mainly related to the mitigation of such toxic effects. Antioxidant drugs do not target any specific biochemical pathways directly but help improve cellular energy metabolism regulation. Due to their non-specific mechanism, these drugs can be used in various PMD with an accumulation of mitochondrial ROS. Several antioxidant drugs have variable degrees of efficacy in terms of longevity and mitigation of oxidative stress in preclinical models of CI defects, pointing at the importance of such treatments in the therapy of PMD [185]. The currently used antioxidant drugs, their clinical uses in MRC diseases, and clinical trials results are discussed below.

#### 3.7.1. Glutathione

Glutathione (GSH; γ-glutamyl-cysteinyl-glycine) is a tripeptide that contains an unusual γ-amide bond; it is a critical intracellular antioxidant agent that is the substrate of several peroxidases, helping to destroy peroxides generated by oxidases. Reduced blood GSH and redox imbalance have been reported in various PMD-patients [186,187]; therefore, supplementation of glutathione precursors may counteract ROS-driven damage. Cysteine donors have received increasing attention as cysteine is the rate-limiting substrate for glutathione biogenesis. However, a 30-day, double-blind, cross-over study providing an oral supplement with a glutathione precursor significantly reduced the oxidative stress biomarkers yet did not modify lactate concentration, clinical scale, or quality of life of the individuals [188]. Beyond its role of GSH precursor, cysteine is required for the 2-thiomodification of mitochondrial tRNAs, which is therefore useful for treating mtDNA mutations affecting mitochondrial transfer tRNA. Supplementation with cysteine, but not N-acetyl-cysteine, partially rescued the mitochondrial translation defect in fibroblasts of patients carrying the m.3243A>G and m.8344A>G mutations, suggesting a possible benefit in a subgroup of patients with impaired mitochondrial translation [189].

#### 3.7.2. Cysteamine

Cysteamine is an amino thiol that is synthesized in mammals, including humans, through the breakdown of Coenzyme A. Cysteamine is an FDA-approved drug for the treatment of cystinosis. This lysosomal storage disease results from defects in the lysosomal cystine transporter (cystinosis), leading to a pathological accumulation of cystine-crystals in lysosomes [190]. Cysteamine exerts its function by entering into the lysosomes where it converts cystine into cysteine and cysteine-cysteamine disulfide, both of which can exit the lysosome [191]. Therefore, cysteamine increases the glutathione precursor cysteine availability, raising the possibility of its repositioning as a drug for PMD. A recent study evaluated cysteamine bitartrate’s therapeutic potential in three different models of mitochondrial disorders: *C. elegans* model of CI defect, *FBXL4* mutant human fibroblast, and zebrafish models of pharmacologically-induced CI and CIV defects [192]. Although a therapeutic potential has been observed, no evident modulation of total glutathione levels was reported, raising concerns about its application in MRC diseases [192].

The microsphere formulation of Cysteamine bitartrate delayed-release (RP103) [193] has been used in a clinical trial. An open-label, dose-escalating study assessing safety, tolerability, efficacy, pharmacokinetics, and pharmacodynamics of RP103 in children affected by inherited PMD was completed in November 2017. RP103 was administered up to 1.3 g/m^2^/day in two divided doses, every 12 h, for up to 6 months. The primary outcome measured focused on changes from baseline in Newcastle Paediatric Mitochondrial Disease Scale Score (NPMDS). Secondary outcomes focused on the measurement of glutathione, lactate, glutathione disulfide, lactate, and evaluation of myopathy by 6 Minute Walk Test. The data analysis is ongoing (ClinicalTrials.gov Identifier NCT02023866).

#### 3.7.3. N-Acetylcysteine

N-acetylcysteine (NAC) also increases glutathione synthesis by increasing cysteine availability, which is, as mentioned above, a rate-limiting substrate for GSH biosynthesis [194]. NAC has been successfully used in a mouse model of ethylmalonic encephalopathy [195]. Ethylmalonic encephalopathy is a severe, fatal disorder caused by mutations in the *ETHE1* gene which encodes a mitochondrial sulfur dioxygenase necessary for the detoxification of sulfide [196]; therefore, mutations in *ETHE1* gene lead to the accumulation of hydrogen sulfide, that is a potent inhibitor of cytochrome c oxidase [197]. Since the supplementation of NAC replenishes the intracellular pool of reduced glutathione, the sulfide is effectively buffered. NAC supplementation is currently used in patients with ethylmalonic encephalopathy [198,199,200], with encouraging results.

#### 3.7.4. Lipoic Acid

Lipoic acid (also called α-lipoic acid) is an essential cofactor covalently bound to several mitochondrial multi-enzymatic complexes, including the ketoglutarate dehydrogenase and pyruvate dehydrogenase [201], involved in energy metabolism. Lipoic acid is also a potent ROS scavenger [202] and antioxidant regenerator in vitro (mainly of CoQ_10_, vitamin C, and glutathione) [203]. However, any increase in radical scavenging activity in vivo is unlikely to be sustained [204], due to the rapid elimination of its free form from cells. Nevertheless, lipoic acid is often administered with other antioxidants to PMD patients [205]. A randomized, double-blind, placebo-controlled, crossover study with 16 patients with mitochondrial diseases demonstrated that the supplementation of lipoic acid combined with creatine monohydrate and CoQ_10_ was able to decrease the levels of oxidative stress markers measured in urine, with parallel amelioration of clinical symptoms [206].

#### 3.7.5. Vitamin C

Limited cases are documenting some improvements with Vitamin C administration, alone or in combination with other drugs. Progressive spasticity in a patient with familial spastic paraparesis and multiple MRC defects was arrested by combined treatment with CoQ_10_, carnitine, vitamin C, and K [207]. Other patients with CIII defect showed mild recovery of some clinical symptoms by combining vitamin C and vitamin K administration [208,209]. However, other patients failed to respond to similar treatment [210].

#### 3.7.6. Vitamin E

The vitamin E-derivative Trolox has been successfully used as ROS scavenger in fibroblasts from patients with CI defect, ameliorating the enzymatic activity’s deficit, supporting evidence that CI expression may be controlled by the cell’s oxidative balance [211]. Moreover, chronic Trolox administration in patients’ fibroblasts with CI defects did restore mitochondrial membrane potential and normalized ER Ca^2+^ uptake without affecting control cell lines [212].

#### 3.7.7. Coenzyme Q_10_

Coenzyme Q_10_ (CoQ_10_, or ubiquinone) is an endogenous, small lipophilic redox-active benzoquinone derivative with an isoprenoid side chain synthesized in every cell apart of erythrocytes. CoQ_10_ is an essential mobile electron carrier, which transfers electrons to mitochondrial respiratory chain CIII from CI and II and the oxidation of fatty acids and branched-chain amino acids. Moreover, CoQ_10,_ in its reduced form (ubiquinol), is an effective lipophilic antioxidant that protects cellular membranes from ROS-mediated oxidation and maintains the vitamin E and vitamin C in their reduced form [213]. CoQ_10_ supplementation may be expected to benefit patients with disorders of the mitochondrial respiratory chain by several mechanisms that are not mutually exclusive. First, it would be useful in patients affected by primary or secondary CoQ_10_ deficiencies, in which there is a pathological reduction of CoQ_10_ due to mutations in genes directly or indirectly involved in the CoQ_10_ biosynthetic pathway, that are, therefore, clinically heterogeneous [214,215]. Second, for the electron carrier properties mentioned above, CoQ_10_ could facilitate electron transport by circumventing a block in the electron transport chain, similar to what has been demonstrated for CIII defect treated with high doses of vitamin C and vitamin K3 [216]. Third, because of its antioxidant properties, CoQ_10_ may accept electrons from disrupted electron transport and reduce ROS formation risk that might cause various cellular damage [217,218]. This is the most general mechanism, potentially applicable to any defect of electron transport [219]. CoQ_10_ is the most common supplement used in PMD patients because it is well tolerated and lacks any chronic side effects. Recent work also provided evidence that CoQ_10_ may act as an enhancer of Parkin-mediated mitophagy flux in trans-mitochondrial cybrids, fibroblasts, and mutant-induced neurons derived from a MERRF patient, with partial improvement of the cellular bioenergetics and pathophysiology [220].

The first paper reporting beneficial effects of CoQ_10_ administration in vivo was published in 1986 and described the effects in five patients with Kearns–Sayre syndrome (KSS). The administration of CoQ_10_ as monotherapy improved abnormal metabolism of pyruvate, as seen by pyruvate/lactate ratio in the cerebrospinal fluid, and NADH oxidation in skeletal muscle, with concomitant amelioration of neurologic symptoms [221]. Since then, many studies have assessed the therapeutic potential of CoQ_10_ administration in patients with mitochondrial respiratory chain disorders. One-year treatment with 120 mg/day of CoQ_10_ in seven patients with KSS and other mitochondrial myopathies with CPEO demonstrated a progressive reduction of serum lactate and pyruvate levels following standard muscle exercise and generally improved neurological functions. Consistent findings on the normalization of pyruvate and lactate levels after exercise have been reported in many clinical studies [222,223,224]. A patient with mitochondrial encephalomyopathy with COX deficiency was treated for two years with a high dose of CoQ_10_ with beneficial effects on pyruvate metabolism and neurological function [225]. Another chronic, 2-year treatment with CoQ_10_ in oral doses of 150–100 mg/day in a patient with KSS syndrome and significantly reduced levels of CoQ_10_ in serum and skeletal muscle biopsy resulted in a marked physical and behavioral improvement. Tremor and ataxia disappeared, but external ophthalmoplegia, retinal degeneration, and cardiac function were unchanged [226]. Treatment with CoQ_10_ improves mitochondrial respiration in skeletal muscle and brain. One study reported that 6 months of treatment with CoQ_10_ (150 mg/day) in 10 patients with mitochondrial cytopathies remarkably improved all brain MRS-measurable variables and muscle rate mitochondrial respiration in all subjects [227]. Supplementation of CoQ_10_ and succinate resulted in clinical improvement of the respiratory function of a patient with Kearns-Sayre and chronic external ophthalmoplegia plus (KS/CEOP). In this case, the patient had virtually no CI activity as a consequence of 4.9 kDa mtDNA deletion; thus, the rationale of the combined treatment was a bypass of the CI defect by feeding the electron transport chain with succinate, plus the electron shuttle CoQ_10_. A direct association between treatment regime and improved clinical status of the patient was documented [228].

In contrast, other studies failed to demonstrate any significant, reproducible, objective clinical improvement following CoQ_10_ administration in a variety of PMD patients [229]. However, the authors reported only a short treatment (2 months). CoQ_10_ treatment also failed to improve ptosis and CPEO [230]. Clinical trials also reported little if no benefit in patients with PMD: a study that enrolled 12 patients with different OXPHOS defects failed to demonstrate any clinical improvement upon CoQ_10_ treatment, regardless of its ability to promote ATP synthetic capacity in peripheral lymphocytes [231]. A randomized, double-blind, cross-over trial was performed in 30 patients with mitochondrial disorders, who received 1200 mg/day CoQ_10_ for 60 days. Although the treatment benefited from aerobic capacity and post-exercise lactate, it did not affect other clinically relevant variables [232]. In a multicenter study, eight patients with different PMD and documented CoQ_10_ defect received 300 mg/day of ubiquinone for 12 months; only subjective improvements on exercise intolerance, fatigue, and stiffness were reported, without any other significant amelioration of other clinical signs [233]. In the same study, CoQ_10_ was also administered to 15 patients with myopathy and normal CoQ_10_ levels in muscle. Only one patient, presenting with encephalomyopathy and an unknown genetic defect, reported subjective improvement of fatigue [233]. A phase 3 trial of CoQ_10_ (ClinicalTrials.gov identifier: NCT00432744) in children with PMD has been designed and implemented; the future outcomes will highlight any therapeutic effects [234].

#### 3.7.8. Idebenone

Since the CoQ_10_ is lipophilic, water insoluble, and poorly absorbed in the gut, novel formulations with improved bioavailability have been developed. Idebenone is an organic molecule of the quinone family, with hydrophilic and redox-active properties, that increases the ATP production, reduces free radicals, inhibits lipid peroxidation, and consequently protects the lipid membranes and mitochondria from oxidative damage [235]. Its pharmacokinetic profile is more favorable than that of its analogue CoQ_10_ [236]. In rats and dogs, the idebenone plasma plateau is reached after 15 min from the administration, with a variable decline of half-life; moreover, idebenone is quickly and homogeneously distributed in the body, but the brain tends to lose its drug content very rapidly [235].

Idebenone is the only EU approved drug for the treatment of LHON. Treatment of fibroblasts from LHON patients with idebenone gave rise to increased CI activity, but yielded contradictory results on mitochondrial respiration, leading to impairment in some cases and stimulation in others [237]. Another study on LHON fibroblasts displayed metabolic alterations that were reversed by idebenone treatments, together with a significant rescue of CI activity [238]. The pharmacological effects of idebenone in retinal ganglion cells (RGC, which are inactive but viable in LHON patients) and in a mouse model of LHON were protective on retinal toxicity and visual impairment induced by CI dysfunction [239]. The first complete randomized, placebo-controlled, double-blind clinical trial in LHON (Rescue of Hereditary Optic Disease Outpatient Study “RHODOS”, ClinicalTrials.gov identifier: NCT00747487) was conducted in 85 LHON patients with m.3460G>A, m.11778G>A, and m.14484T>C mutations. This study demonstrated the safety and well tolerability of idebenone (900 mg/day for 24 weeks) and reported amelioration of the visual outcome in a subgroup of patients [240]. Another randomized, double-blind placebo-controlled intervention study investigated the red–green (protan) and blue–yellow (tritan) color contrast sensitivity in 39 LHON patients, demonstrating significant protection from loss of color vision in subjects receiving idebenone for 6 months [241]. A clinical trial consisting of a single visit follow-up observational study in a subset of patients enrolled in the RHODOS study (RHODOS-OFU, ClinicalTrials.gov identifier: NCT01421381) demonstrated that the beneficial effect of idebenone treatment persisted despite discontinuation of therapy [242]. Additional studies are required to confirm these initial observations.

While use for LHON patients is well described, the exact mechanism is still undeciphered. However, beneficial effect of idebenone administration was described in an old adolescent patient suffering from an infantile-onset neurodegenerative disorder with severe cerebellar atrophy, epilepsy, dystonia, optic atrophy, and peripheral neuropathy, diagnosed with an homozygous stop mutation in Thioredoxin 2 (*TXN2*). TXN2 is a small mitochondrial redox protein essential for controlling the homeostasis of mitochondrial reactive oxygen species; based on the established defect in ROS regulation, TXN2 patient was treated with Idebenone (900 mg/day) in a compassionate use. During the 4 months follow-up period the, patient showed an improvement of feeding behavior (less tube feeding required), a considerable weight gain and increased physical capacity [243].

Idebenone has also been used to treat *OPA1*-dependent Dominant Optic Atrophy. Dominant optic atrophy (DOA) arises from heterozygous mutations in the *OPA1* gene that promotes fusion of the inner mitochondrial membrane and plays a role in maintaining ATP levels. Patients display optic disc pallor, RGC loss, and bilaterally reduced vision [244]. A randomized, placebo-controlled trial of idebenone at 2000 mg/kg/day in *Opa1* mutant mice with visual loss revealed limited therapeutic effects on RGC dendropathy and visual functions and showed a detrimental effect of idebenone in wild-type mice [245]. Nevertheless, patients’ results are more encouraging: a pilot study on seven DOA patients documented encouraging results after 1-year of idebenone administration, with some improvement of visual function [246]. A recent retrospective cohort study investigated the effect of off-label idebenone administration on visual outcome in a DOA group of 87 patients, demonstrating that the treatment was significantly associated with stabilization/recovery of visual acuity [247].

#### 3.7.9. MitoQ

MitoQ is a CoQ_10_ analogue that contains the antioxidant quinone moiety covalently attached to a lipophilic triphenylphosphonium cation (TPP^+^), specifically designed to be accumulated by mitochondria in vivo, driven by the plasma- and mitochondrial-membrane potential [248]. To enter mitochondria, alkyl triphenylphosphonium cations first bind to the inner membrane’s outer surface, then permeate the phospholipid bilayer’s hydrophobic potential energy barrier, before binding to the inner surface of the membrane [249]. Once imported into mitochondria, nearly all the molecule is adsorbed into the IMM matrix surface, where it is continuously recycled to the antioxidant quinol form by the succinate-CoQ reductase [249,250]. However, MitoQ does not work as an electron carrier because it is a poor substrate for CI, CIII, and electron-transferring flavoprotein (ETF): quinone oxidoreductase (ETF-QOR) [250]. The selective accumulation of MitoQ prevents mitochondrial oxidative damage far more efficiently than untargeted antioxidants, although an intact mitochondrial membrane potential is required for its efficacy [251]. In vivo studies assessed that MitoQ can be safely administered for long term treatments [252,253]. Therefore, it has been developed as a pharmaceutical compound by Antipodean Pharmaceuticals Inc. and tested in few clinical trials to evaluate the beneficial effect of its antioxidant properties. The PROTECT study (ClinicalTrials.gov identifier: NCT00329056) evaluated the effect of MitoQ administration on the progression of Parkinson’s Disease, which showed no significant improvement compared to the placebo group [254]. Some encouraging results have instead been obtained in age-related vascular dysfunction [255]. Although MitoQ is the most extensively studied mitochondria-targeted antioxidant in several disease contexts ranging from diabetes [256] to ageing [257] and heart failure [258] among others, MitoQ efficacy has never been evaluated in patients with PMD.

### 3.8. Redox-Active Molecules 

#### 3.8.1. EPI-Molecules

Modifications of the redox head and lipid tail of the CoQ_10_ molecule accomplished by Enns and co-workers [259] led to new experimental, redox-active molecules. Such new drugs, including the EPI-743, EPI-A0001, EPI-589 work as pro-oxidant, electron shuttles, and also display antioxidant properties. Importantly, the chemical modifications of the quinone ring, i.e., the substitution of the two methoxy groups with two methyl groups, significantly increased the redox properties of EPI-743, which undergo oxidation-reduction at a redox potential offset by −75 mV compared to CoQ_10_ and idebenone [259]. The changes at the isoprene tail significantly reduce these three molecules’ lipophilicity, thus raising their bioavailability.

EPI-743 (Vatiquinone) is a drug belonging to the class of para-benzoquinones, a group of potent cellular oxidative stress protectants. EPI-743 targets the enzyme NADPH quinone oxidoreductase 1 (NQO1), increasing the biosynthesis of glutathione and modulating the redox control of metabolism [259]. It is an orally bioavailable molecule that can efficiently cross the blood–brain barrier [259]. The first clinical trial in 2011 enrolled 14 participants, who were selected based on two criteria: (I) genetically confirmed mitochondrial disease; and (II) possibility of end-of-life care starting within 90 days. All but one patient had an encephalomyopathy phenotype. Subjects were treated with EPI-743 orally or via gastrostomy tube for 12 weeks in a subject controlled, open-label study. Two patients died; the twelve survivors showed a modified disease progression, with a significant improvement of quality of life, brain imaging parameters, and clinical in >90% of the cases [259]. A prospective single-arm subject-controlled trial of EPI-743 was conducted in 2012 in children with genetically confirmed Leigh syndrome, at least moderately severe disease and MRI confirmation of necrotizing encephalopathy [260]. Subjects were treated for six months, with 100 mg of EPI-743 three times daily orally or via a gastrostomy tube. The clinical outcome showed that all children demonstrated arrested of the disease progression and/or reversal [260]. Analysis of blood samples in other children with mitochondrial encephalopathy showed EPI-743 administration’s ability to restore reduced glutathione pools [261]. A recent case report documented the visible improvement of a pediatric patient with Leigh syndrome due to a mutation in the mitochondrially encoded ND3 gene treated with EPI-743. She was the only child surviving after four years of age, suggesting that EPI-743 could modify the natural course of the syndrome and contribute to the patient’s long-term survival [262]. In a small open-label trial, EPI-743 arrested disease progression and reversed vision loss in most treated patients with LHON, suggesting that the previously described irreversible priming to retinal ganglion cell loss may be reversed by EPI-743 administration [263]. Other clinical trials evaluating the efficacy of EPI-743 in mitochondrial disease are still ongoing: one study has recruited 31 patients with Leigh syndrome to evaluate the long-term safety and neurodevelopmental effects of EPI-743 administration the dose of 15 mg/kg, up to a total 200 mg three times daily. The estimated primary completion date is December 2021 (ClinicalTrials.gov Identifier: NCT02352896). Another non-randomized, double-blind, placebo-controlled, cross-over study has finished recruiting children aged 2–11 with PMD in 2019. The primary outcome measures the effects of EPI-743 on quality of life. Secondary outcome measures include various biochemical, imaging, and clinical abnormalities (ClinicalTrials.gov Identifier: NCT01642056). Other molecules of the EPI series could be applied in the treatment of PMD due to OXPHOS defect, such as EPI-A0001 and EPI-589 although they have not been tested yet. EPI-A0001 (α-tocopheryl quinone) is a potent antioxidant, that has been tested for the treatment of Friedreich ataxia [264]. Only one double-blind, randomized, placebo-controlled, 28-days trial of two doses of EPI-A0001 in 31 patients reported encouraging results in terms of improvement of neurological function (ClinicalTrials.gov Identifier: NCT01035671). However, no other further studies have been reported. EPI-589, also known as (R)-troloxamide quinone, is expected to increase the reserves of antioxidant molecules, but to date, there are no published data regarding its mechanism of action. It is currently used for the treatment of ALS and in a clinical trial for Parkinson’s Disease.

#### 3.8.2. JP4-039

The affinity of the antibiotic Gramicidin S for the bacterial membrane has inspired the chemical structure of the JP4-039 molecule, a new, mitochondrial-targeted antioxidant drug [265]. JP4-039 displayed electron scavenger properties in animal models and in several tumor cell lines, as well as to improve mitochondrial respiration and scavenge ROS in *ACAD9*- [266] and in Very Long-Chain Acyl-CoA Dehydrogenase (*VLCAD*)- mutant fibroblasts [267]. Similar results have been reported in *ETHE1* and *MOCS1* mutant cell lines, in which JP4-039 treatment did increase the oxygen consumption rate, ATP production, and decrease superoxide levels. Preliminary pharmacokinetics after intravenous administration suggested fair tissue distribution, including in the brain, opening future perspectives for mitochondrial neurological disease therapies.

#### 3.8.3. KH176

The ROS-Redox modulator KH176 was developed by the optimization of the Trolox-derivatives molecules [268]. KH176 has a dual effect: (I) it successfully reduces cellular ROS levels, and (II) it protects against redox perturbation by targeting the thioredoxin/peroxiredoxin system. The mechanism of action of KH176 requires its conversion into the quinone metabolite KH176m [268]. KH176 could counteract the ROS production and mitigate the altered cellular redox state in cellular models of CI defects [268]. The therapeutic efficacy of KH176 was tested in preclinical models of PMD. Long-term KH176 treatment ameliorated the clinical phenotype and the brain microstructural coherence of the CI-deficient *Ndufs4* ko mouse model [269,270]; however, no further improvement was observed with combined treatment with the PPAR agonist clofibrate [270]. A Phase 1 clinical trial in healthy adult male volunteers deemed that KH176 is well tolerated up to single doses of 800 mg and multiple doses of 400 mg b.i.d. and has a pharmacokinetic profile supportive for a twice-daily dose (ClinicalTrials.gov Identifier NCT02544217) [271]. Phase 2, double-blind, randomized, placebo-controlled, single-center, two-way cross-over trial has also been performed [272] (The KHENERGY STUDY - ClinicalTrials.gov Identifier NCT02909400). This study recruited patients with m.3242A>G mutation and aimed to explore the effects of treatment with KH176 for 4 weeks on clinical signs and symptoms and biomarkers of PMD and evaluate the KH176-related safety and pharmacokinetics. Results confirmed that KH176 was well tolerated and appeared safe at the 100-mg twice a day dose regimen; a significant improvement of the patients’ overall mental health status was also documented [272]. Recently, KH176 (*Sonlicromanol*, developed by the biopharmaceutical company Khondrion, The Netherlands), received a rare pediatric disease (RPD) designation from the United States Food and Drug Administration (FDA), for the treatment of patients with MELAS syndrome [273]. Sonlicromanol is currently in Phase IIb clinical development (The KHENERGYZE Study, ClinicalTrials.gov Identifier: NCT04165239).

#### 3.8.4. SKQ1

The mitochondria-targeted antioxidant 10-(6′-plastoquinonyl)-decyl-triphenyl-phosphonium (SKQ1) is a cationic plastoquinone derivative containing a positively charged phosphonium connected to plastoquinone by a decane linker. The antioxidant activities of mitochondria-targeted cationic plastoquinone derivatives (SKQs) are accomplished in two different ways: (I) by preventing peroxidation of cardiolipin [274] (mediated by quinol moieties) and (II) by fatty acid cycling, resulting in mild uncoupling that inhibits the formation of ROS in mitochondrial State IV (mediated by cation moieties) [275]. SKQ1 can effectively mitigate the oxidation induced either by hydrogen peroxide or by organic hydroperoxide in vitro [276]. SKQ1 has mainly been tested in several pathological cellular and pre-clinical models in which ROS-mediated mitochondrial dysfunction and cell death play a crucial role, such as Alzheimer’s Disease [277,278], multiple sclerosis [279], and Parkinson’s Disease [280]. In contrast, only one work tested its efficacy in a PMD model [281]. Shabalina and co-workers reported that chronic administration of SKQ1 to the *Mutator* mouse ameliorated mitochondrial ultrastructure in several tissues and significantly improved age-related phenotypic features, including the occurrence of hair loss, kyphosis, loss of estrus cycle, body weight loss, reduced lipid stores, hypothermia, immobility, and torpor-like states. Most importantly, SKQ1 administration significantly increased the lifespan of the *Mutator* mice [281]. However, increased oxidative damage has not been observed in the mtDNA *Mutator* mice (as reviewed by Edgar and Trifunovic [282]).

### 3.9. Pharmacological Modulation of Mitochondrial Dynamics

Mitochondria are highly dynamic organelles that undergo coordinated cycles of fission and fusion, referred to as “mitochondrial dynamics”, to maintain their shape, distribution, and size [283]. Mitochondrial shape and mass are finely tuned by the activity of the pro-fusion proteins Mitofusin 1 (MFN1) and Mitofusin 2 (MFN2)—acting on the outer mitochondrial membrane (OMM)—and optic atrophy protein 1 (OPA1)—acting on the inner mitochondrial membrane (IMM)—plus the antagonist action of pro-fission proteins, such as dynamin-related protein 1 (DRP1) and mitochondrial fission 1 protein (FIS1) [284]. OPA1 is a multitasking GTPase with a total of eight long and short isoforms, which are involved in two independent mechanisms: (I) tighten of mitochondrial cristae of the IMM [285], that favors MRC supercomplexes assembly and optimizes mitochondrial respiration [286] (II) elongation of the mitochondrial network, promoting mitochondrial fusion [287]. Modifying these processes may benefit different PMD [98].

Genetic disorders of mitochondrial dynamics comprise defects of mitochondrial fusion triggered by mutations in *MFN2* or *OPA1*, exhibiting as Charcot–Marie–Tooth type 2A and autosomal dominant optic atrophy, respectively [288,289,290], and impaired mitochondrial fission caused by mutations in *DRP1* [291] and *MFF* [292]. The observation that the overexpression of OPA1 increased respiratory efficiency by stabilizing the respiratory chain SC [287] suggested that moderate overexpression of OPA1 could be beneficial in MRC defects models. Significant amelioration of mitochondrial encephalopathy and myopathy was obtained in a mouse model of COX defect crossed with a transgenic *Opa1* mouse model [293]. Furthermore, *Opa1* overexpression also prevented kidney focal glomerulosclerosis in the *Mpv17* ko mouse [294]. The recent discovery of chemical modulators of mitochondrial fusion (M-hydrazone) and fission (MDIVI-1 and P110) may represent a therapeutic option for OXPHOS defect [295,296,297].

Mitochondrial dynamics could be indirectly modulated, targeting the cytoskeleton organization, which has a significant role in supporting the mitochondrial network [298]. Recently, the *Escherichia coli* protein toxin called Cytotoxic Necrotizing Factor 1 (CNF1), which acts on the Rho GTPases regulators of the actin cytoskeleton [299], was tested in OXPHOS deficient patients‘cells [300]. CNF1 effectively induced mitochondrial elongation, rescuing the wild-type-like mitochondrial morphology and increasing the ATP content in fibroblasts derived from a MERRF patient with m.8344A>G mutation [300]. Further studies are needed to assess the potential use of these drugs on preclinical models of PMDs.

### 3.10. Pharmacological Protection of Cardiolipin

Among experimentally new drugs for PMD, there is Elamipretide (also known as MTP-131, SS-31, and Bendavia), a small aromatic-cationic tetrapeptide (D-Arg-dimethylTyr-Lys-Phe-NH_2_) that readily penetrates cell membranes in a non-energy requiring and non-saturable manner, and transiently localizes to the IMM, where reversibly binds to cardiolipin [301]. Cardiolipin is a unique phospholipid that is only located on the IMM and plays essential structural roles in modulating the IMM curvature leading to cristae formation and organizing the electron transport chain complexes into SC to facilitate optimal electron transfer and energy production. Cardiolipin also plays a role in anchoring cytochrome c to the inner membrane facilitating electron transfer from CIII to CIV [302]. When oxidized, cardiolipin participates in cell death. It is highly vulnerable to oxidative damage because it contains many unsaturated fatty acids and is located close to reactive oxygen species’ production site. Oxidation of cardiolipin leads to the disruption of intimal microregions and the loss of membrane curvature and cristae [303]. Elamipretide binds selectively to cardiolipin via electrostatic and hydrophobic interactions and protects it from oxidation, keeping mitochondrial cristae, promoting oxidative phosphorylation, and inhibiting mitochondrial permeability transition pore opening [205]. The idea is that elamipretide restores energy production, reduces the production of reactive oxygen species, and ultimately increases the energy supply to affected cells and organs. It has been shown that elamipretide consistently improves mitochondrial, cellular, and organ function in both in vitro and in vivo disease models for which mitochondrial dysfunction is understood to be an essential component, including cardiovascular, renal, metabolic, skeletal muscle, neurodegenerative, and mitochondrial genetic disease [304,305,306,307,308].

Elamipretide is metabolized via sequential C-terminal degradation to the tripeptide M1 and the dipeptide M2. The apparent plasma half-life (t½) of M1 was comparable to that of elamipretide, whereas t½ of M2 was longer than that of elamipretide. elamipretide and its metabolites are excreted primarily through the kidneys. Elamipretide was initially used in preclinical models to treat ischemia/reperfusion injury, a common complication of interventional procedures for acute myocardial infarction and coronary bypass surgery [309].

As of November 2017, the U.S. FDA Office of Orphan Products Development has granted Orphan Drug Designation to Stealth’s investigational drug candidate, elamipretide, to treat patients with primary mitochondrial myopathy (MM). SPIMM-301 was a Phase 3, multicentered, double-blind, parallel-group, placebo-controlled trial followed by an open-label treatment extension (ClinicalTrials.gov Identifier: NCT03323749). It evaluated the efficacy and safety of elamipretide over 32 weeks in 218 patients, ages 16 to 80, with MM. Enrolled subjects received in Part 1 single daily 40 mg/mL subcutaneous injections of fixed doses of elamipretide/placebo for up to 24 weeks; in Part 2 received single daily 40 mg/mL subcutaneous injections of fixed doses of elamipretide for up to 144 weeks. The trial was conducted at 28 clinical sites across North America, Europe, and Australia. The primary endpoints assessed efficacies were the 6-min walk test (6MWT) and the Primary Mitochondrial Myopathy Symptom Assessment (PMMSA) Total Fatigue Score. The 6MWT measures the distance an individual can walk over a total of 6 min on a hard, flat surface. PMMSA is a patient-reported outcome tool developed by Stealth in which individuals with PMM report their fatigue, muscle weakness, and other symptoms on a scale from 1 (least severe) to 4 (most severe). Safety results showed that treatment with elamipretide was well-tolerated with most adverse events mild to moderate in severity, but it did not produce significant improvements in 6MWT and PMMSA assessments [310].

Elamipretide is now in late-stage clinical studies in other three PMD: Barth syndrome (ClinicalTrials.gov Identifier: NCT03098797), Leber’s hereditary optic neuropathy (ClinicalTrials.gov Identifier: NCT02693119), as well as a clinical study in dry age-related macular degeneration (AMD) with non-central geographic atrophy (ClinicalTrials.gov Identifier: NCT03891875).

### 3.11. Pharmacological Modulation of Autophagy

Autophagy is an evolutionarily conserved process that degrades cargoes-like aggregate-prone proteins, pathogens, damaged organelles, and macromolecules via delivery to lysosomes, to warrant cellular quality control. Targets for degradation are first encircled into specific, double-membrane structures termed autophagosomes, whose formation (phagophore), elongation, and closure are controlled by autophagy-related (ATG) proteins [311]. Autophagosomes are fused with lysosomes to generate autophagolysosomes that carry out the degradation of the substrates. Stimulation of autophagy has been proposed as a therapeutic approach to target and eliminate dysfunctional mitochondria. The most widely used inhibitor of (macro) autophagy is rapamycin, which acts by blocking the target of rapamycin (mTOR) complex 1 (mTORC1) [312]. Johnson and co-workers first published the results of a chronic treatment of a PMD mouse model with the mTOR inhibitor Rapamycin in 2013. The authors reported a significant delay in the disease progression and fatal outcome of the *Ndufs4* ko mouse [313]. These results were further confirmed in other preclinical models of OXPHOS, including (I) a muscle-specific *Cox15* ko mouse [314], (II) an *ND2*-deficient *Drosophila* model of LS [315], (III) iPSCs-derived neurons carrying a mutation in the *MT-ATP6* gene [316], and (IV) the gas-1 (fc21) nematodes [317]. These encouraging results led to developing a clinical study (ClinicalTrials.gov Identifier: NCT03747328) in four MELAS patients treated with *Everolimus*, a rapamycin analogue. Patients’ derived primary fibroblasts showed improvement of mitochondrial morphology, membrane potential, and replicative capacity [318]. Recently, *Everolimus* was used to treat two children affected by Leigh disease or MELAS. The latter failed to respond and died of progressive disease 10 months after starting the treatment [319], whereas the child with Leigh syndrome improved health status. Brain MRI reduced the bilateral signal hyperintensity in thalami and brainstem after 6 months of treatment. Further improvements were documented after 19 months of treatment, being the patient able to walk independently with a slightly ataxic gait, and with no longer required of tracheostomy and gastrostomy. However, although these data support the idea that rapamycin may be useful in several PMD, others recently reported that rapamycin treatment exacerbated the disease progression in mice with CoQ_10_ deficiency [320] and failed to rescue the cerebral pathological features of TwKO^astro^ mice [59], indicating that not all metabolic defects may benefit from rapamycin therapy. Moreover, mTORC1 inhibitors are linked to immunosuppressive outcomes [321], and it is currently unknown whether this effect could be detrimental for PMD patients in the long-term. 

### 3.12. Bypassing cI-cIII-cIV Defects with Alternative Enzymes

A recent therapeutic strategy concerns the possibility to by-pass OXPHOS defects by using the alternative enzymes NADH dehydrogenase/CoQ_10_ reductase (NDI1), plant alternative NADH dehydrogenases (NDH-2), and CoQ_10_/O_2_ alternative oxidase (AOX). AOX and NDI1 are single-peptide enzymes present in yeast, plants, and lower eukaryotes where they act as alternative components of the respiratory chain. NDI1 substitutes CI in yeast, where it transfers electrons to CoQ_10_ and regenerates the NAD^+^ pool, while AOX bypasses CIII and CIV by accepting electrons from CoQ_10_. In contrast to *Saccharomyces Cerevisiae* NDI1, which enzymatically competes with endogenous CI [322], plant alternative NDH-2 naturally coexists with endogenous CI and supports the oxidation of NADH only in specific physiological conditions [323], e.g., when CI is metabolically inactive, or the concentration of matrix NADH exceeds a certain threshold. It should be noted that such alternatives electron transfer activities are not linked to a proton pumping across the inner mitochondrial membrane. Expression of these enzymes has been used to bypass CI deficiency in *Drosophila melanogaster* [324], combined CI-III-IV deficiencies in *ρ*^0^ mouse cells [325], CIII-IV deficiencies in human cells [326] and *Drosophila melanogaster* [327], raising the possibility to use these genes to treat OXPHOS-related disorders. Adeno-associated virus (AAV) expressing *NDI1* (AAV-NDI1) was shown to protect retinal ganglion cells (RGCs) in a rotenone-induced murine model of LHON, significantly reducing RGC death by 1.5-fold and optic nerve atrophy by 1.4-fold and considerably preserving retinal function [328]. Recently, the effects of the expression of NDI1 in vivo have been tested in a mouse model of Leigh syndrome due to the lack of the 18-KDa complex I subunit Ndufs4 [329]. McElroy and co-workers generated a mouse that conditionally expressed the yeast *NDI1*, while the *Ndufs4* was lost, specifically in the brain. NDI1 expression was sufficient to dramatically prolong lifespan without significantly ameliorate the ataxic phenotype [329].

Interestingly, the authors demonstrated that mitochondrial CI activity in the brain supports organismal survival through its NAD^+^ regeneration capacity, while optimal motor control requires the bioenergetic function of mitochondrial CI [329]. When transgenic *Ciona intestinalis AOX* mice were crossed with CIII-deficient *Bcs1l^p.S78G^* knock-in mice—a model of GRACILE syndrome (growth retardation, aminoaciduria, cholestasis, iron overload, lactic acidosis, and early death) [330,331], with multiple visceral manifestations and premature death—AOX expression was able to increase lifespan, prevent lethal cardiomyopathy, and ameliorate renal and cerebral manifestations. On the contrary, when the transgenic *AOX* mouse was crossed with the *Acta*-*Cox15* ko model, the double ko-*AOX* mutants showed a decreased lifespan and a substantial worsening of the myopathy compared to the ko alone. Decreased ROS production in ko-*AOX* versus ko mice led to impaired AMPK/PGC-1α signaling and PAX7/MYOD-dependent muscle regeneration, blunting compensatory responses [332].

Recently, the two different mitochondrially-targeted *NDH*-*2* (AtNDA2 and AtNDB4) from *Arabidopsis thaliana* (At) were used to bypass the OXPHOS defect in human CI deficient fibroblasts and reduce oxidative stress [333].However, a competition between AtNDA2 and endogenous CI for NADH oxidation was reported in control cell lines [333], raising some concern about its potential therapeutic application for human PMD.

## 4. Precision Medicine Approaches for PMD Caused by mtDNA Defects

### 4.1. Pre-Implantation Therapies to Prevent the Transmission of mtDNA Mutations

Each mammalian cell contains numerous copies of mtDNA. The coexistence of mutated and wild-type mtDNA molecules is called heteroplasmy, the percentage of which can range from negligible values to 100%. Heteroplasmy allows detrimental mutations to persist, and most importantly, to be transferred to the next generation. MtDNA molecules segregate unequally during primordial germline development. Upon oocyte maturation, such segregated pools of mtDNA expand within each egg cell. In the case of an asymptomatic mother carrying heteroplasmic mutations in her germline, the heterogeneous population of oocytes could develop offspring with vastly varying levels of heteroplasmy [334]. Therefore, pathogenic mutations of mtDNA are maternally transmitted [335] although a rare, paternal inheritance was debated in the last years, and recently reviewed by Wei and Chinnery [336]. Heteroplasmy is well tolerated until the percentage of the mutation (i.e., mutational load) exceeds a certain threshold, often greater than 60% mutated mtDNA, beyond which bioenergetic defect manifests, mainly in high energy demanding tissues. No effective therapies for mtDNA-linked disease exist, and several techniques can prevent the transmission of pathogenic mutations.

#### 4.1.1. Pre-Implantation Genetic Diagnosis

Pre-implantation genetic diagnosis is a preventive approach that still represents families’ best option with a known story of mtDNA mutations [337]. Pre-implantation diagnosis is an in vitro fertilization (IVF)-based approach in which the fertilized egg harboring the pathogenic mtDNA mutation is cultured until the stage of 6–8 cells [338] or blastocyst [339] and then biopsied for genetic analysis before implantation. However, the pre-implantation diagnosis has some limitations: (I) it will only benefit women who have low levels of mtDNA mutations in oocytes [340] and, (II) it assumes that the diagnosed heteroplasmy level is representative of that of the entire embryo and would not change over time.

#### 4.1.2. Mitochondrial Donation: Maternal Spindle Transfer

**Figure 3:**
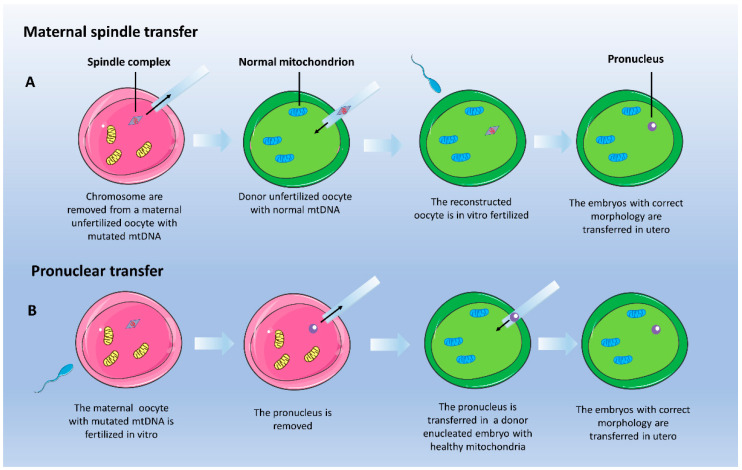
Mitochondrial donation (MD). (**A**) Maternal spindle transfer (MST) and (**B**) pronuclear transfer (PNT) represent the two principal strategies to prevent transmission of mtDNA disease. The techniques forecast the removal of nuclear genetic material from patient and maternal oocytes pre- or post-fertilization. The maternal genetic material is then transferred to enucleated oocyte or zygote from the donor, thereby generating an embryo characterized by the parental nuclear genetic material and by healthy mitochondria from the donor.

Recently, mitochondrial replacement or mitochondrial donation (MD) has been proposed as a potential method for preventing transmission of mutated mtDNA from the mother to the offspring, by replacing the mitochondria in the oocytes of carrier women. The most recently exploited MD reproductive technologies include maternal spindle transfer (MST) [341] and pronuclear transfer (PNT) [342]. This technology is legally approved for use in the U.K., but the governments of Australia and Singapore are in the process of formal discussions aimed at MD legalization. However, the current state of MD-relevant activity and regulation remains largely elusive in many countries; please refer to [343] for a detailed overview. MST is a complex technique that involves the transfer of nuclear genetic material between a patient’s egg with mutated mtDNA to an enucleated donor’s unfertilized metaphase II oocyte with healthy mitochondria. MST generates an oocyte with a patient’s nuclear DNA devoid of mutated mtDNA. MST is then followed by intracytoplasmic sperm injection (ICSI) and in vitro embryo culture (Figure 3A). A proof-of-principle use of MST to prevent the transmission of mutated mtDNA molecules was first reported in rhesus macaques by the group of Mitalipov in 2009 [344]. In that case, MST and subsequent ICSI resulted in the birth of healthy offspring (named Mito and Tracker) with undetectable levels of spindle donor’s associated mtDNA [344]. The same group then translated this technique to human oocytes, and similar fertilization and blastulation rates between MST and control groups were observed, suggesting that embryo development was not compromised [345]. MtDNA analysis revealed that all examined spindle transfer zygotes and cleaving embryos contained more than 99% donor mtDNA. Similar outcomes were observed in 87% of embryonic stem (ES) cell lines established from spindle transfer blastocysts, regardless of donor mtDNA. However, a reversal of mtDNA haplogroup from donor to maternal mtDNA in a limited number of ES clones was reported, for which a mechanistic explanation based on replicative advantages conferred by some D-loops polymorphisms were proposed [345]. It should be noted that a further mtDNA analysis on those and others ES cell lines [346] raised some concerns about the evidence provided by Kang and co-workers [345]. At the American Society for Reproductive Medicine (ASRM) annual meeting 2016, Dr. John Zhang, New Hope Fertility Center of New York City reported the outcome of the use of MST in a woman carrying mtDNA mutation of Leigh syndrome (8993 T>G), which resulted in the birth of an healthy baby with less than 10% mutated mtDNA in tissues tested 2 days after the birth [347]. A case report was published in 2017 [348].

#### 4.1.3. Mitochondrial Donation: Pronuclear Transfer

The pronuclear transfer (PNT) involves a first step of in vitro fertilization of the patient’s oocyte, followed by the removal of the diploid nucleus, which is then transferred into a donor’s enucleated zygote with healthy mitochondria (Figure 3B) [340]. PNT was proposed in 2005 to prevent transmission of mtDNA disease in the mito-mouse, a model that accumulates large-scale mtDNA deletions [349]. In this elegant paper, second polar bodies were used as biopsy samples to diagnose mtDNA genotypes of mito-mouse zygotes. Nuclear transplantation was carried out from mito-mouse zygotes to enucleated normal zygotes and was shown to rescue all of the F(0) progeny from the expression of respiration defects throughout their lives [349]. In a second report published in 2010, abnormally fertilized human zygotes were used, and reconstructed embryos developed following PNT showed the capacity to reach the blastocyst stage [340]. In 2016, the first preclinical evaluation of PNT using normally fertilized human embryos was reported [350]. Since the techniques used these studies [340] were not tolerated by normally fertilized zygotes, an alternative approach was developed, that significantly improved the efficient development to the blastocyst stage. This was based on performing the pronuclear transplantation immediately after completing meiosis rather than before the first mitotic division. Following this optimization, mtDNA carryover was reduced to less than 2% in the majority (79%) of PNT blastocysts. The study reported low levels of mtDNA carryover in PNT embryos and observed a reversion to the maternal haplogroup in a limited number of hESC clones derived from PNT inner cell mass embryos [350]. In conclusion, although PNT can reduce the risk of mtDNA disease, it may not guarantee prevention [350].

### 4.2. Personalized Therapies for mtDNA Disorders

#### 4.2.1. Delivery of Nucleic Acids to the Mitochondria

Mutations in mitochondrial genes or mitochondrial tRNAs are associated with a variety of maternally inherited neuromyopathies. An effective therapy would imply delivery therapeutic genes or tRNAs to the mitochondrial matrix, where mtDNA resides.

Nucleic acid delivery into the mitochondrion has been attempted using liposome-based nanocarriers such as Mito-Porter [351,352] and dequalinium-based liposome-like vesicles (DQAsomes)-transfection system [353], or by RNA Import Complex [354]. Mito-Porter is a liposome-based carrier that introduces macromolecular cargos into mitochondria via membrane fusion. The authors provided evidence of nucleic acid delivery into the mitochondrial matrix either in isolated rat liver mitochondria and in living, intact cells [351]. As proof of principle, this method was further exploited to deliver wild-type mitochondrial pre-tRNA^Phe^ to decrease the mutation rate of tRNA^Phe^ in mitochondria of the patient’s cell with a G625A heteroplasmic mutation in the tRNA^Phe^ of mtDNA, with a significant correction of the mutation rate [355]. Similarly, a therapeutic correction of ND3 mutant fibroblasts’ mitochondrial respiration was obtained by reintroducing the wt mRNA of ND3 via Mito-Porter [356].

DQAsomes are the prototype for all mitochondria-targeted vesicular pharmaceutical nanocarrier systems. First described in 1998, they have been successfully explored to deliver DNA and low-molecular-weight molecules to mitochondria within living mammalian cells [357].

The RNA Import Complex (RIC) is a multi-subunit protein complex from the mitochondria of the Kinetoplastid protozoon *Leishmania tropica* that induces the transport of tRNA across natural and artificial membranes [358]. Observing that Kinetoplastid protozoa have evolved specialized systems for importing nucleus-encoded tRNAs into mitochondria, the RIC was used to import endogenous cytosolic tRNAs, including tRNA^Lys^, and restored mitochondrial function in wild-type, MERFF, and KSS cybrids [354].

However, no effective treatment was translated into animal models despite this evidence of delivering the exogenous nucleic acids into the cellular mitochondria.

#### 4.2.2. Heteroplasmic Shift

A second strategy to correct mtDNA mutations is based on the disruption of mutant molecules using selective nucleases to shift the heteroplasmy level below the critical, pathological threshold. To this aim, several approaches based on the following tools have been developed in the past years and are discussed below: (I) restriction enzymes; (II) antisense oligonucleotides; (III) molecular scissors; (IV) DddA.

The very first work carried out by Tanaka and co-workers in 2002 [359] implied the delivery of the restriction endonuclease SmaI into the mitochondria of cybrids to specifically degrade mtDNA in which the pathogenic 8993T>G mutation was present, creating a specific SmaI-restriction site [359]. SmaI specifically degraded mutant mtDNA, with consequent repopulation of mitochondrial genome by wild-type molecules, and subsequent restoration of intracellular ATP level and mitochondrial membrane potential [359].

Heteroplasmy shifting has also been achieved using antisense oligonucleotides in cybrids containing a heteroplasmic mtDNA deletion [360]. In this case, anti-replicative RNA molecules were designed and transfected into a cybrid cell line derived by a KSS patient’s fibroblasts carrying an mtDNA deletion involving 65% of the mtDNA molecules. The anti-replicative effect of the RNA oligoribonucleotides complementary to the mutant mtDNA region specifically reduced the proportion of pathological mtDNA population, shifting the heteroplasmy level from 65% to 50% [360].

The Clustered Regularly Interspaced Short Palindromic Repeats (CRISPR)/Cas9 technology has been largely used for nuclear gene editing; however its application for the editing of the mitochondrial genome appears highly challenging mainly due to sub-efficient delivery of guide RNA and Cas9 enzyme complexes into mitochondria. However, in 2015, Jo and colleagues successfully manipulated the mtDNA with the CRISPR/Cas9 technology [361]. Despite the lack of MTS peptides, the authors showed that the flagged Cas9 localized into the mitochondria, while the gRNAs allowed specific depletion of targeted portions of mtDNA but not degradation of the entire molecule. This point was difficult to reconcile with the fact that mtDNA behaves as a unit [362].

A recent preprint study reported the editing of the *MT-ND4* gene accomplished by targeting the guide RNA to an RNA transport derived stem loop element (RP-loop), while expressing the Cas9 enzyme with a mitochondrial localization sequence [363]. However, due to the controversial nature of mammalian mitochondrial RNA import [364], the use of the CRISPR/Cas9 application for mtDNA editing is still debated.

Very recently, adeno-associated viral vectors (AAVs) were used to deliver molecular scissors (i.e., ZFNs, mitochondrial-targeted (mt) TALENs and mtZFNs) in vivo, to destroy mutated mtDNA selectively. Using the first available mouse model of heteroplasmic mitochondrial disease, bearing the point mutation m.5024C>T in mitochondrial tRNA^ALA^ (mt-tRNA^ALA^) [365], both TALENs and mtZFNs were able to reduce the mtDNA heteroplasmy with the concomitant rescue of molecular and biochemical phenotypes [366,367].

However, the approaches described so far cannot introduce specific nucleotide changes in mtDNA and cannot be applied to homoplasmic mtDNA mutations because they would destroy all mtDNA copies. To overcome those issues, Mok et al. has recently set up a precision genome editing strategy. They obtained outbreaking results using the interbacterial toxin “double-stranded DNA deaminase toxin A”, or DddA, encoded by *Burkholderia cenocepacia,* which catalyzes the deamination of cytidines within dsDNA [368]. Engineered split-DddA halves are inactive until simultaneously carried on the target DNA by adjacently bound programmable DNA-binding proteins. The fusions of the split-DddA halves, transcription activator-like effector (TALE) array proteins, and an uracil glycosylase inhibitor generated the RNA-free DddA-derived cytosine base editors (DdCBEs), which allows guided CG-to-TA changes in mtDNA without the need of double-strand breaks. This technique can potentially correct pathogenic mutations on mtDNA with high levels of purity and specificity [368]. Despite these findings looking extremely innovative, additional research aimed to improve the delivery of DdCBEs in vitro and in vivo is essential for exploring their therapeutic potential.

#### 4.2.3. Allotopic Gene Expression

Allotopic gene expression is a method to overcome the mtDNA mutations by re-expressing the missing mtDNA-encoded protein from the nucleus. In this case, an engineered nuclear version of a mitochondrial gene encodes a protein that can be imported into the mitochondria due to an MTS presence. This approach was used to deliver the protein product of the MT-ATP6 gene to the mitochondria in cybrids containing the m.8993T>G mutation [369]. Such allotopic expression significantly improved the cell growth in selective medium and the ATP synthesis [369]. Similarly, allotopic expression of the MT-ND4 gene effectively prevented blindness in a rat model of LHON [370]. Consequently, a human clinical trial of gene therapy to treat LHON has been carried out. Although there was weak evidence of allotopic expression, Guy et al. reported the amelioration of visual acuity in the injected eyes [371]. Another clinical trial (ClinicalTrials.gov identifier NTC01267422) in which nine patients affected by LHON carrying the G11778A mutation were treated with a single dose (5 × 109 vg/0.05 mL) of rAAV2-*ND4* reported no adverse effects. In six out of nine patients, the injected eyes’ visual acuity improved by at least 0.3 log MAR after nine months of follow-up. The visual field was enlarged, but the retinal nerve fiber layer remained relatively stable. The visual acuity improved, and the visual field was enlarged nine months after treatment, while other parameters were unchanged [372]. A third open-label phase I/II clinical trial (ClinicalTrials.gov identifier NCT02064569) investigated both safety and preliminary efficacy of a rAAV2/2-*ND4* in four dose-escalation cohorts. The treatment resulted in safe with mild adverse effects. Six out of 14 patients manifested a clinically relevant improvement in the best-corrected visual acuity. Taken together, these results suggest the possible use of gene therapy for LHON.

#### 4.2.4. Mitochondrial Augmentation Therapy

Recently, Minovia Therapeutics developed a novel form of cellular therapy called Mitochondrial Augmentation Therapy (MAT). Its rationale is based on the capability of exogenous mitochondria to enter cells in culture [373,374], bringing their own genetic material, augmenting endogenous mitochondrial function, and content and fusing with the endogenous mitochondrial network. The mitochondrial uptake process was termed mitochondrial augmentation and is currently proposed for Kearns–Sayre syndrome (KSS) and Pearson Syndrome. KSS is a progressive retinitis pigmentosa and external ophthalmoplegia occurring at childhood due to de-novo mtDNA deletions [13]. Other systems may be involved over time, including hearing, heart, skeletal muscles, central nervous system, endocrine glands, and kidneys [13]. Pearson Syndrome is a rare disorder affecting the bone marrow and exocrine pancreas [14], also characterized by de-novo mtDNA deletion [15]. MAT approach enriches hematopoietic stem cells (HSCs) with healthy mitochondria before transplantation in patients. MAT involves a series of complex steps: first, mitochondria are extracted from white blood cells derived from the patient’s mother (confirmed non-deleted); in turn, stem cells are collected from the patient, who receives treatment with *Neupogen* (filgrastim, by Amgen) to boost the production of white blood cells in the bone marrow, and with *Mozobil* (plerixafor, by Sanofi-Aventis) to mobilize the hematopoietic stem cells containing the CD34 protein marker into the bloodstream. The healthy mitochondria are then introduced into patient-derived stem cells and given back to the patient by intravenous infusion. A first human study was performed on three children with Pearson syndrome under a compassionate use program. Results were reported at the “Targeting Mitochondria Conference 2019” and at “Mitochondrial Medicine 2019 Meeting”. Mitochondrial augmentation therapy improved the in vitro PS-derived HSC function, the metabolic determinants, the aerobic capacity, and the quality of life of the treated patients. The same company also promoted an open-label study (ClinicalTrials.gov identifier NCT03384420) to assess the MAT’s safety and therapeutic effects. Recruitment of seven children is now in progress to analyze any treatment-related adverse events at one year, and any improvement in the quality of life, as assessed by the International Pediatric Mitochondrial Disease Scale.

Moreover, promising results of the first MAT treatment on a KSS patient were recently reported. The 14-year-old patient underwent leukapheresis, and positively selected CD34^+^ cells were augmented with maternal mitochondria before infusion (2 × 10^6^ cells/kg). Then, the patient was followed for clinical and metabolic parameters. Before MAT, she weighed only 19 kg, she could not sit, walk, express words, and experienced 1 or 2 seizures a week. Seven months after treatment, she gained weight, she could reach objects, sit independently, walk with assistance, and express herself in short sentences. Seizures were resolved 4 months after treatment. Her normalized functional score on the International Pediatric Mitochondrial Disease Score improved from 91% to 57%. Also, the ATP content of the peripheral blood lymphocytes was increased. Those impressive improvements of her physiological and metabolic status make MAT a potential therapy for KSS [375].

## 5. Precision Medicine Approaches for PMD Caused by Nuclear Defects

### 5.1. Gene Therapy Approaches

Gene therapy is the most straightforward option for treating disease caused by a single recessive genetic defect. Re-expressing the wild-type form of a mutated gene, or other therapeutic genes, using appropriate viral vectors is an attractive strategy, currently exploited for disorders affecting a single organ. In fact, while the expression of an ectopic gene in the whole body is still unfeasible, specific critical cells or tissue can be targeted with currently available technologies. AAVs are emerging as a suitable delivery method because they are not associated with any disease in humans or animals and remain episomic in the cells for a prolonged time, thus reducing the risk of insertional mutagenesis [376]. Moreover, the availability of several serotypes allows tissue-specific targeting [377]. Potential pitfalls concern the limited cloning capacity, the difficulty in achieving therapeutic expression levels in several tissues—especially skeletal muscle, due to is abundance and distribution- and the brain, due to the low blood–brain-barrier (BBB) penetrance. One of the first preclinical gene therapy studies applied to a mitochondrial myopathy was performed using the AAV2 carrying the *ANT1* cDNA in the *ANT1* ko mouse model. *ANT1* encodes the mitochondrial adenine nucleotide translocator, an integral IMM protein that forms homodimers or tetramers [378], acting as electrogenic pumps that export ATP out across the IMM in exchange for cytosolic ADP [379]. Mutations in *ANT1* lead to MM with progressive external ophthalmoplegia (PEO), caused by paralysis of the extraocular eye muscles [380]. AAV-*ANT1* transduction resulted in long-term stable expression in muscle precursor cells and differentiated muscle fibers. The transgenic ANT1 protein was targeted into the IMM, formed a functional ADP/ATP carrier, increased the mitochondrial export of ATP, and reversed the histopathological changes associated with the MM [381]. Furthermore, the efficacy of AAV-mediated gene therapy has been confirmed in several mouse models of mitochondrial disorders, including the models for hepato-cerebral forms of severe mtDNA depletion syndrome [57] and Leigh syndrome [382].

### 5.2. Liver Transplantation

Liver transplantation (LT) is a feasible approach to treat PMD, mainly affecting the single organ [383]. LT has been performed in individuals with hepatocerebral forms of mtDNA depletion syndromes, which frequently progress to liver failure, as those due to mutations in *MPV17* gene [384]. However, LT outcome has not been satisfactory, since almost fifty percent of the transplanted patients died in the post-transplantation period due to multiorgan failure. For this and other reasons, LT in mitochondrial hepatopathies remains controversial. LT is not recommended in patients with disorders characterized by a rapid progression of neurological manifestations, such as Alpers–Huttenlocher syndrome, but it may be beneficial in patients with an acceptable quality of life. LT may also differentially affect survival, e.g., patients with mtDNA depletion syndromes caused by mutations in *DGUOK* gene—encoding the mitochondrial deoxyguanosine kinase, which phosphorylates purines to the corresponding nucleotides in the mitochondrial nucleotides salvage pathway—show lower survival than those of patients with other PMD. Although a recent retrospective study on 12 PMD patients receiving LT confirmed these findings [385], others suggest that, even in the presence of neurological MRI findings, but in the absence of significant neurological symptoms, LT represents a viable option in DGUOK-deficient patients [386].

### 5.3. PMD Characterised by Systemic Accumulation of Toxic Compounds

#### 5.3.1. Application of Gene Therapy Protocol

AAV therapy has been successfully achieved by targeting the missing gene to the liver when the underlying disease mechanism is linked to toxic compounds’ systemic accumulation. This is the case of the ethylmalonic encephalopathy caused by mutations in the *ETHE1* gene encoding for a mitochondrial sulfur dioxygenase involved in the detoxification pathway of hydrogen sulfide (H_2_S) [387]. Mutations in *ETHE1* lead to systemic accumulation of H_2_S, which acts as a potent inhibitor of complex IV [197], leading to the onset of the phenotype (for a detailed description of the H_2_S pathway, see Viscomi, Bottani et al., 2015 [388]). The re-expression of the *ETHE1* gene by a hepatotropic AAV2/8 serotype restored the missing enzymatic activity in the liver, with a significant clearance of H_2_S from the bloodstream, a major amelioration of the phenotype and prolonged lifespan of the *Ethe1* ko mice [389]. Similarly, systemic accumulations of thymidine and deoxy uridine, which interfere with mtDNA replication and lead to mitochondrial dysfunction were corrected by hepatotropic AAV2/8 vector carrying the human *TYMP* in a mouse model of MNGIE [390]. The nucleoside reduction achieved by this treatment prevented deoxycytidine triphosphate (dCTP) depletion, which is the limiting factor affecting mtDNA replication in this disease [390].

AAV treatment may be useful also in mitochondrial neurodegenerative disorders: it was reported that the administration of AAV9 carrying the human *NDUFS4* partially rescued the phenotype of Ndufs4 ko mice only when simultaneously administered systemically and intracranially. AAV9 serotype did not efficiently cross the BBB, and mainly targeted glial cells when injected intracranially in new-borns. Interestingly, newly engineered serotypes AAV-PHP.eB and AAV-PHP.S showed great promises in their efficiency to transduce the central and peripheral nervous systems, crossing the BBB [391].

#### 5.3.2. Application of Liver Transplantation Protocol

Like the gene therapy approach, the liver is an attractive target for pathologies triggered by systemic accumulation of toxic compounds. LT is proposed as an alternative approach to treat mitochondrial neurogastrointestinal encephalopathy (MNGIE) disease. MNGIE is caused by a deficiency of thymidine phosphorylase (TP) due to mutations in the nuclear gene *TYMP* [392]. TP is a cytosolic enzyme catalyzing the first step of thymidine (dThd) and deoxy uridine (dUrd) catabolism. Mutations in TYMP lead to the accumulation of dThd and dUrd systemically, which induce an imbalance of the cytosolic nucleotide pool. Because the mitochondrial nucleotide pool relies, in part, on nucleotides imported from the cytosol, an imbalanced cytosolic nucleotide pool lead to an imbalanced mitochondrial nucleotide pool, which ultimately has mutagenic effects on mtDNA, resulting in depletion, multiple deletions, and point mutations causing progressive mitochondrial deficiency and organ failure [392,393,394]. LT rapidly normalized serum levels of toxic nucleosides in a 25-year-old MNGIE patient, and his conditions were stable after 400 days of follow-up [395]. LT also resulted in an effective option to treat ethylmalonic encephalopathy due to mutation in *ETHE1* gene [396,397], since the replaced organ can substitute the deficient ETHE1 enzyme and clear the toxic H_2_S that accumulate in this disorder, constituting a feasible therapeutic option in patients. Patients showed progressive improvement of the neurological functions and normalization or amelioration of the biochemical abnormalities [396,397]. However, the decision to perform LT remains difficult because neurological manifestations may worsen despite their absence before the transplant [398].

#### 5.3.3. Cell Replacement

Cell therapy consists of using cells or cell-based products to replace dead or defective cells to restore the function of the affected tissue(s) [399]. Again, this approach has been proposed to treat MNGIE. While TP is not expressed in all tissues, cellular and plasmatic dThd and dUrd levels are in equilibrium among all body compartments. Therefore, replacing the lost enzyme in a circulating form—i.e., in the blood cells—should favor the catabolism of the toxic metabolites in plasma, thus clearing these freely diffusible substrates from the tissue compartments, normalizing the cellular nucleotide pools, and preventing further mtDNA damage.

Cell replacement therapies may offer a permanent cure to MNGIE. They comprise (I) Allogeneic Hematopoietic Stem Cell Transplantation (AHSCT) and (II) erythrocyte-encapsulated thymidine phosphorylase (EE-TP). Clinical and biochemical improvements following AHSCT have been reported in MNGIE patients [400,401]. Although AHSCT corrects biochemical abnormalities and improves gastrointestinal symptoms, the procedure is risky for subjects in poor medical conditions, as many MNGIE patients are. Since transplant-related morbidity and mortality increases with the progression of the disease and the number of comorbidities, MNGIE patients should be submitted to AHSCT when they are still relatively healthy, to minimize the complications of the procedure [401].

EE-TP consists of the ex-vivo encapsulation of *Escherichia coli* TP within the patient’s autologous erythrocytes using a reversible hypo-osmotic dialysis process. Once inside the erythrocyte, the encapsulated enzyme catalyzes the deoxyribonucleosides’ metabolism to the specific products thymine and uracil, which then exit the erythrocyte and flow into their physiological metabolic pathways. Recently, three adult MNGIE patients received escalating intravenous doses of EE-TP. EE-TP was well tolerated, and reductions in the disease-associated plasma metabolites, thymidine, and deoxy uridine were observed. Clinical ameliorations, including weight gain and improved disease scores, were observed in two patients, suggesting that EE-TP can reverse some aspects of the disease [401]. Advantages of the EE-TP are the prolonged circulatory half-life of the enzyme and the minimization of the immunogenic reactions compared to those frequently observed in enzyme replacement therapies administered by the conventional route [401].

### 5.4. Molecular Bypass Therapy in Disorders of mtDNA Instability

Syndromes characterized by mtDNA instability are usually due to mutations in nuclear genes involved either in the mtDNA replication machinery or deoxynucleotide triphosphate (dNTP) metabolism consequently affecting OXPHOS activities.

From the clinical perspective, these diseases are characterized by disorders that range from severe infantile hepatocerebral encephalopathy to childhood-onset myopathy or adult-onset PEO [402]. The TK2 gene provides an example. The protein product of this gene is a deoxyribonucleoside kinase with mitochondrial localization that specifically phosphorylates thymidine, deoxycytidine, and deoxyuridine. This enzyme is required for mtDNA synthesis. Recessive mutations in the human TK2 gene are responsible for the myopathic form of the mitochondrial depletion/multiple deletions syndrome [403]. Mitochondrial dNTPs pools are supplied either by *de novo* synthesis and import from the cytosol or by the mitochondrial deoxyribonucleoside salvage pathway [404]. Supplementation of the missing or insufficient dNTP may bypass the block restoring the deoxynucleotides triphosphate (dNTP) pools. Molecular bypass therapy (MBP) with deoxypyrimidine monophosphates (dCMP and dTMP) or substrate enhancement therapy with deoxypyrimidine nucleosides (dC and dT) were tested on the Tk2 knock-in (ki) mouse model in the early pre-symptomatic, but biochemically affected, stage [405]. Treatment with dCMP and dTMP raised dTTP concentrations, increase levels of mtDNA, ameliorated the defects of MRC enzymes, and significantly prolonged the lifespan (from 13 to 34 days) in a dose-dependent manner [406,407]. Late treatment of 29-day-old mice was ineffective.

Similar strategies were then extended in different models of PMD. The mtDNA depletion in human fibroblasts mutated in the DGUOK gene was ameliorated by the supplementation of deoxyguanosine [404], and similarly, pyrimidine and purine nucleoside administration, but not the corresponding monophosphate nucleotides, adjusted the mtDNA depletion induced by ethidium bromide in human RRM2B-mutant cells [408]. Similarly, deoxycytidine and tetrahydro uridine were also able to prevent mtDNA depletion in a cell model of the same syndrome [408]. Recently a mutant *dguok* zebrafish line was developed using CRISPR/Cas9 mediated mutagenesis; *dguok^−/−^* fishes have significantly reduced mtDNA levels compared to the wild-type counterpart. In contrast with previous cell culture studies, when supplemented with only one purine nucleoside (dGuo), mtDNA copy number in both mutant and wt juvenile animals was significantly reduced, possibly because of nucleotide pool imbalance. However, a significant increase in liver mtDNA was documented in adult *dguok*^−/−^ zebrafish when supplemented with both purine nucleosides. [409].

Recently, an open-label study showed the results of deoxynucleoside monophosphates and deoxynucleoside administration under a compassionate program to 16 early-onset TK2-patients. Prolonged survival and improvement of motor abilities compared to untreated patients were recorded. Four of 5 patients who required enteric nutrition were able to discontinue using the feeding tube; and 1 of 9 patients who required mechanical ventilation became able to breathe independently. Out of 8, 3 non-ambulatory patients recovered the ability to walk. Out of 5, 4 patients with enteric nutrition discontinued the use of the feeding tube. Out of 9, 1 patient who required mechanical ventilation became able to breathe independently. Although diarrhea was the most common side effect manifested, discontinuation of the therapy was not necessary. Among 12 other TK2 patients treated with deoxynucleoside, two adults developed elevated liver enzymes normalized following discontinuation of therapy [410].

## 6. Future Perspectives

### 6.1. Fetal Gene Therapy

Current prenatal genetic technology can diagnose rare genetic diseases as early as the 12th week of pregnancy by chorionic villous sampling or from the 16th week by amniocentesis. Advances in fetal imaging and minimally invasive surgical equipment have also led to the development of interventional techniques for prenatal treatment of several fetal structural defects, such as congenital diaphragmatic hernia, myelomeningocele, pulmonary sequestration, hydrothorax, urinary tract obstruction, fetal tumours and others [411]. Also, intrauterine access to the fetal circulation through the umbilical vein is a well-described procedure, used to perform ultrasound-guided fetal blood or platelet transfusions, which are currently the standard of care in cases of fetal anemia and thrombocytopenia [412]. The option of fetal intervention is offered in specialized centers to reduce infant mortality and/or morbidity compared to postnatal treatment and, for each type of procedure, parents are extensively informed about the potential benefits balanced against the risks, which are mainly fetal death, miscarriage or preterm delivery.

For inherited genetic diseases, In-Utero Gene Therapy (IUGT) offers the potential of prophylaxis against early, irreversible, and lethal pathological change [413]. From a technical perspective, potential genetic therapeutic agents can be delivered to the fetus through infusion into the umbilical vein or via direct injection into the fetal organs. The rationale for IUGT is essentially based on the hypothesis that anticipating the treatment during fetal life could prevent or mitigate irreversible pathological changes associated with rare genetic disorders and improve clinical outcomes compared to postnatal therapy. Possible advantages of IUGT that may overcome some of the limitations encountered in postnatal gene therapy include: (i) the small fetal size (i.e., smaller area to be treated), (ii) the tolerogenic fetal immune system, (iii) the presence of highly proliferative and accessible stem/progenitor cells in multiple organs and, (iv) the ability to treat diseases in which irreversible pathological molecular and metabolic changes begin before birth [414].

Fetal therapeutic interventions could be especially useful for early-onset PMD in which mitochondrial dysfunction begins before birth, as the GRACILE syndrome. GRACILE syndrome initially develops with intrauterine growth retardation. A fatal lactic acidosis arises in the new-borns, often accompanied by nonspecific aminoaciduria, cholestasis, iron overload, and liver dysfunction [415].

Similarly, mitochondrial dysfunction likely starts before birth in SURF1-associated Leigh syndrome. Shreds of evidence suggest that *SURF1* mutations lead to metabolic impairments in neural progenitor cells (NPCs), which cannot switch from glycolytic to OXPHOS metabolism, with subsequent aberrant proliferation and insufficient support for neuronal morphogenesis [416]. A similar neuronal impairment was reported in a *SURF1* ko pig model [417]. Cerebral organoids from LS patients carrying *MT-ATP6* mutations also showed defective corticogenesis and suggest pre-natal impairment [418]. These findings suggest that OXPHOS defect could impair the NSC cellular metabolism in the early phase of the development leading to the onset of the neurological phenotypes; so prenatal intervention for pediatric PMD may be crucial for amelioration of the clinical course of the disease.

Fetal gene therapy may provide an alternative therapeutic approach for inherited diseases leading to early death or lifelong irreversible damage. Due to the lack of PMD-specific, fetal therapeutic approaches, curative strategies proposed for other genetic diseases should be considered. A recent study investigated the efficacy of human survival motor neuron (hSMN) gene expression after IU delivery in SMA mouse embryos. In the first part of the research report, authors showed that IU-intracerebroventricular injection of adeno-associated virus serotype-9 (AAV9)-EGFP led to an extensive expression of EGFP protein in different parts of the CNS with a significant number of transduced NSCs. SMA mouse fetuses receiving a single i.c.v. injection of a single-stranded or self-complementary AAV9-SMN vector extended their lifespan of 93 (median of 63) or 171 (median 105) days. Both muscle pathology and motor neuron survival improved upon treatments, with slightly better results from scAAV administration [413].

Additional evidence on the safety and efficacy of IUGT in a mouse model of neuronopathic Gaucher’s disease were recently provided [419]. Fetal intracranial injection of an AAV9 carrying the curative gene improved neuronal inflammation and spectacularly increased the mice’s overall survival. Of note, neonatal treatment did not achieve the same results of fetal therapy.

Maternal safety is a critical consideration in any fetal therapy. Possible maternal exposure to the viral vectors infused into the fetus should be considered in IUGT. Contact with the viral particles may result in maternal immune responses to the capsid protein or the recombinant protein, although the latter is unlikely, as the mother should already be producing—and therefore be tolerant to—the protein missing in the fetus.

Therapeutic viruses, including the AAV vectors, may undergo random integration in fetuses after IUGT; however, do evidences of germline integration have been reported. New technologies with more specific gene editing will possibly minimize off-target events of IUGT in future.

Despite promising preliminary results of IUGT, more experimental evidence on animal models is needed to demonstrate a significant improvement in the pathological hallmarks and the clinical course of the disease. Clinical trials involving human pregnancies would subsequently need to be setup, ensuring accurate monitoring of adverse events and long-term postnatal clinical follow-up. Randomized controlled trials on IUGT versus postnatal treatment may not be realistic due to the rarity of the investigated diseases and ethical concerns that may arise from offering randomization for conditions associated with a very severe if not lethal outcome. Also, rare genetic disorders currently lack any recommended screening policy in the general low-risk population. Therefore, most of the patients currently receiving a prenatal diagnosis of PMD have experienced a previously affected child’s birth. In these cases, prenatal treatment parents could be offered within an experimental trial, after extensive counselling on the uncertainties regarding the clinical results and after discussion of other management options, which would be termination of pregnancy or postnatal therapy. It could be anticipated that the recruitment rate in such a context is likely to be extremely low. However, multicenter collaborative efforts may allow to collect and analyze a meaningful number of cases using a pre-defined shared protocol, to provide reliable information on the effects of IUGT on long-term postnatal development of infants affected by rare genetic disorders.

Ethical issues of fetal gene therapy have been reviewed by MacKenzie and collaborators [420]. Fetal treatment pivots on the concept of non-directive advising, in which the choices of no treatment and exploratory therapy—with all the conceivable risks and benefits—are rationalized without the physician’s individual inclination. Rigorous preclinical trials and multidisciplinary debates will continue to advance the frontiers of fetal therapy, while these and other concerns deserve a continuous discussion [420].

### 6.2. Metabolic Rewiring

Pharmacological modulations of neuronal morphogenesis and neuronal maturation of the immature precursors, the neural stem cells (NSCs) have been proposed as an attractive therapeutic opportunity to treat several neurological diseases. NSCs require a metabolic shift towards oxidative phosphorylation during the process of neuronal differentiation [421]. Therefore, OXPHOS defects may inhibit this shift impairing neuronal differentiation and driving neural stem cells (NSCs) to a proliferative and less differentiated state [416]. We recently tested inductors of oxidative metabolism (developed and patented by Professional Dietetics, IT) composed of TCA cycle intermediates, specific amino acids, and co-factors that helped enhance mitochondrial function in different in vitro and in vivo models. Supplementing wild-type NSC-culture medium with these compounds during the differentiation phase enhanced the metabolic shift towards OXPHOS and mitochondrial function of mouse and human NSCs, improving their full differentiation capacity [422]. Neurons derived from treated-NSC changed the fission–fusion processing resulting in a mitochondrial elongated phenotype; moreover, the activation of the mTORC1 pathway with subsequent significant increase of ATP production was reported. Also, the antioxidant defense system was also triggered by the increase of the NRF2 gene expression [422]. We observed similar metabolic and molecular changes in vivo, where they counteracted the pathological mitochondrial dysfunction occurring during the aging process. Three-month oral administration of metabolic inductor PD-0E7 (Professional Dietetics, IT) to the Senescence Accelerated Mouse-Prone 8 (SAMP8) mice significantly improved the sarcopenia and cognitive decline, enhancing oxidative metabolism by inducing mitochondrial biogenesis and increasing respiratory efficiency [423]. In particular, we documented a strong shift toward oxidative, COX-positive fibers and a general increase of the MRC enzymatic activities in the skeletal muscle of the 12-month-old PD-0E7-supplemented mice, which may explain the preserved physical endurance of the treated SAMP8 mice [423]. Also, the Opa1 isoforms were significantly increased in the skeletal muscle, as shown by western blot analysis, and this might support the improved stabilization of the CIII holocomplex into SCs that was detected by blue native gel electrophoresis (BNGE) analysis [423]. To note, the preserved cognitive function observed in the treated mice correlate with enhancement of the hippocampal mitochondrial proteostasis and with the upregulation of PITRM1, a mitochondrial matrix enzyme that digests the mitochondrial targeting sequences (MTS) and the mitochondrial fraction of amyloid beta [424,425].

Although the molecular mechanisms at the basis of the improvement of mitochondrial functions by such metabolic modulators were not fully elucidated, it is plausible that supplementation of TCA cycle intermediates and amino acids would feed the anaplerotic flux sustaining mitochondrial energy production. In support of this, it has recently been demonstrated that anaplerosis is protective in OXPHOS-deficient neurons with disruption of the *MFN2* gene, and that genetic blockage of the anaplerotic pathway further exacerbated the neuronal degeneration [426]. To date, triheptanoin is the only reported example of anaplerotic treatment in patients with very-long-chain acyl-CoA dehydrogenase (VLCAD) deficiency [61]. Studies of the effects of anaplerotic substrates in PMD should be encouraged.

## 7. Conclusions

The extreme clinical, genetic, and biochemical variability of PMD coupled with the low number of patients and the frequent lack of adequate preclinical models have limited identifying useful clinical outcomes and the development of effective therapy.

The enhancement of mitochondrial function and ATP production through the pharmacological stimulation of mitochondrial biogenesis, mitophagy, dynamics, and ROS detoxification using antioxidants may represent a general strategy to alleviate or at least partially corrected different clinical outcomes. Although these strategies do not solve the problem at the root, these are, in principle, adaptable to a large group of mitochondrial diseases and could help improve patients’ everyday quality of life.

**Figure 4:**
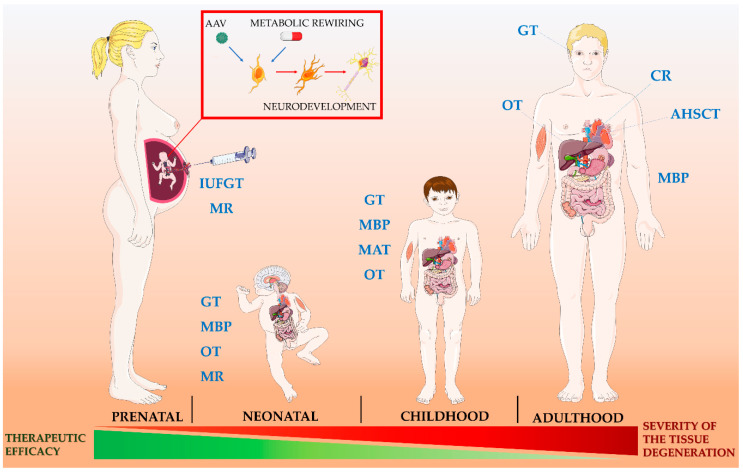
Severity of the tissue degeneration increases with age, impacting the efficacy of therapeutic interventions. IUFGT: In utero fetal gene therapy; MR: metabolic rewiring; MBP: Molecular Bypass Therapy; MAT: Mitochondrial Augmentation Therapy; GT: Gene Therapy; OT: Organ Transplantation; CR: Cell Replacement; AHSCT: Allogeneic Hematopoietic Stem Cell Transplantation.

Conclusive cure for mitochondrial disease could be achieved by a precision medicine strategy that considers individual variability in genes, age, sex, stage of the disease, and tissues compromised for each patient (Figure 4). Although organ transplantation was already used successfully, cell replacement and gene therapy are still far to become routine for mitochondrial disease due to technical and regulatory reasons. However, given the recent exciting progress in gene editing and fetal surgery, we expect steps forward in the coming decades.

## Appendix A

**Table A1:** Summary of the therapeutic approaches discussed in this manuscript, with a distinction between those that have been tested in pre-clinical models and those that have already been used in clinical practice. This table is restricted to therapies used to treat PMD and does not include others that, although cited in the text, have not been tested in PMD models/patients.

Therapy	Model	Study	Ref.
Strategies to increase ATP levels			
Febuxostat plus inosine	patients with homoplasmic mtDNA mutation	clinical	[65]
	patient with mitochondrial diabetes with heteroplasmic mutation in tRNA leucine 1	clinical	[65]
Pharmacological stimulation of mitochondrial biogenesis			
AICAR	mouse models of COX defects	pre-clinical	[74]
	mouse model Cox10-Mef2c-Cre	pre-clinical	[82]
	CI deficient cells (NDUFS2, NDUFS4, NDUFAF4, C20ORF7, FOXRED1, NDUFA12L)	pre-clinical	[83]
Bezafibrate and other PPAR agonists	MRC-deficient patients’ fibroblasts	pre-clinical	[87]
	SCO2 mutant fibroblasts	pre-clinical	[88]
	DNM1L mutant cells	pre-clinical	[89]
	mouse models of COX defects	pre-clinical	[74]
	*Deletor* mouse	pre-clinical	[90]
	*Mutator* mouse	pre-clinical	[91]
	patients with MM	clinical	[92]
NAD^+^ precursors	*Sco2* ko mouse	pre-clinical	[81]
	*Deletor* mouse	pre-clinical	[106]
	GBA-PD *Drosophila melanogaster*	pre-clinical	[107]
	*Ndufs4* ko mice	pre-clinical	[108]
Niacin	patients with MM	clinical (NCT03973203)	[109]
I-BET 525762A	cybrids carrying 3796A>G mutation	pre-clinical	[110]
Polyphenols and other Pharmacognostic Products			
Resveratrol	fibroblasts from MT-TL1, MT-TK, MT-ATP6-patients	pre-clinical	[125]
Curcumin	LHON patients	clinical (NCT00528151)	unpublished
Pharmacological modulation of the NO/cGMP/PKG pathway			
L-arginine and L-citrulline	MELAS patients	clinical (open-label trial)	[162,164,168]
	MELAS patients	clinical (open-label trial)	[165]
	various PMD patients	clinical (NCT02809170)	unpublished
	MELAS patients	Phase-1 clinical trial NCT03952234	unpublished
PDE5 inhibitors	NPCs with homoplasmic mutation in MT-ATP6	pre-clinical	[176]
	LHON	clinical (case report)	[179]
Antioxidant			
Glutathione	MM patients	clinical (double-blind cross-over study)	[188]
	fibroblasts of patients carrying the m.3243A>G and m.8344A>G mutations	pre-clinical	[189]
Cysteamine	*C. elegans* model of CI defect, FBXL4 mutant human fibroblast, and zebrafish models of pharmacologically induced CI and CIV defects	pre-clinical	[192]
Cysteamine bitartrate delayed-release (RP103)	PMD paediatric patients	clinical (NCT02023866)	unpublished
N-acetylcysteine	*Ethe1* mouse model	pre-clinical	[195]
	*ETHE1* patients	clinical (compassionate use)	[198,199,200]
Lipoic acid and CoQ10	PMD patients	clinical (randomized, double-blind, placebo-controlled, crossover study)	[206]
Vitamin C and K	PMD patients	Clinical	[208,209,210]
Vitamin E	fibroblasts from patients with CI defect	pre-clinical	[211,212]
Coenzyme Q_10_	MERRF cells	pre-clinical	[220]
	KSS patients	clinical	[221]
	KSS patents and other MM with CPEO	clinical	[222,223,224,226,228,230]
	patient with mitochondrial encephalomyopathy with COX deficiency	clinical	[225]
	patients with mitochondrial cytopathies	clinical	[227]
	PMD patients	clinical	[229]
	patients with different OXPHOS defects	clinical	[231]
	PMD patients	randomized, double-blind, cross-over trial	[232]
	patients with different PMD; 15 patients with MM	clinical (multicenter study)	[233]
	PMD paediatric patients	clinical (NCT00432744)	[234]
Idebenone	fibroblasts from LHON patients	pre-clinical	[237,238]
	mouse model of LHON	pre-clinical	[239]
	85 LHON patients with to m.3460G>A, m.11778G>A, and m.14484T>C mutations	clinical (“RHODOS” study, NCT00747487)	[241]
	subset of LHON patients from RHODOS study	clinical (RHODOS-OFU, NCT01421381)	[242]
	patient with TXN2 mutation		[243]
	*Opa1* mutant mice	pre-clinical	[245]
	seven DOA patients	clinial	[246]
	87 DOA patients	clinical (retrospective cohort study)	[247]
Redox-Active Molecules			
EPI-743	PMD patients	clinical	[259]
	patients with Leigh syndrome	clinical (prospective single-arm subject-controlled trial)	[260]
	children with mitochondrial encephalopathy	Clinical	[261]
	one patient with Leigh syndrome due to ND3 mutation	Clinical	[262]
	patients with LHON	clinical (open-label trial)	[263]
	patients with Leigh syndrome	clinical (NCT02352896)	unpublished
	PMD paediatric patients	clinical (NCT01642056)	unpublished
JP4-039	ACAD9- VLCAD-, ETHE1 and MOCS1 mutant fibroblasts	pre-clinical	[266,267]
KH176	cellular models of CI defects	pre-clinical	[268]
	*Ndufs4* ko mouse model	pre-clinical	[269,270]
	patients with m.3242A>G mutation	clinical (KHENERGY STUDY, NCT02909400)	[272]
	patients with MELAS	clinical (KHENERGYZE Study, NCT04165239)	[273]
SKQ1	*Mutator* mouse	pre-clinical	[281]
Pharmacological modulation of mitochondrial dynamics			
Cytotoxic Necrotizing Factor 1 (CNF1)	MERRF fibroblasts	pre-clinical	[300]
Pharmacological protection of cardiolipin			
Elamipretide	patients with primary mitochondrial myopathy (MM)	clinical (NCT03323749)	[310]
	Barth syndrome	clinical (NCT03098797)	unpublished
	LHON patients	clinical (NCT02693119)	unpublished
	age-related macular degeneration (AMD) with non-central geographic atrophy	clinical (NCT03891875)	unpublished
Pharmacological modulation of autophagy			
Rapamycin	*Ndufs4* ko mouse	pre-clinical	[313]
	muscle-specific *Cox15* ko mouse	pre-clinical	[314]
	ND2-deficient Drosophila model of LS	pre-clinical	[315]
	MT-ATP6-mutant, iPSCs-derived neurons	pre-clinical	[316]
	gas-1 (fc21) nematodes	pre-clinical	[317]
	mice with CoQ_10_ deficiency	pre-clinical	[320]
	TwKO^astro^ mice	pre-clinical	[59]
Everolimus (rapamycin analogue)	MELAS patients	clinical (NCT03747328)	[318]
	children affected by Leigh disease or MELAS	Clinical	[319]
Bypassing cI-cIII-cIV defects with alternative enzymes			
NDI1	CI deficiency in *Drosophila melanogaster*	pre-clinical	[324]
	mouse model of LHON	pre-clinical	[238]
	mouse model of Leigh syndrome	pre-clinical	[239]
AOX, NDI1	*ρ*^0^ mouse cells	pre-clinical	[325]
AOX	CIII-IV deficiencies in human cells	pre-clinical	[326]
	CIV deficient *Drosophila melanogaster*	pre-clinical	[327]
	*Bcs1l^p.S78G^* knock-in mice	pre-clinical	[330,331]
	*Acta-Cox15* ko model	pre-clinical	[332]
NDH-2	human CI deficient fibroblasts	pre-clinical	[333]
Personalized therapies for mtDNA disorders			
Mitochondrial donation: maternal spindle transfer	legally approved for use in the U.K	clinical	[343]
	rhesus macaques	pre-clinical	[344]
	woman carrying mtDNA mutation of Leigh syndrome (8993 T>G)	clinical	[347,348]
Mitochondrial donation: pronuclear transfer	*mito*-mouse	pre-clinical	[349]
Delivery of nucleic acids to the mitochondria	patient’s cell with a G625A heteroplasmic mutation in the tRNA^Phe^	pre-clinical	[355]
	ND3 mutant fibroblasts	pre-clinical	[356]
	MERFF and KSS cybrids	pre-clinical	[354]
Heteroplasmic shift	various cells with heteroplasmic mutation	pre-clinical	[359,360,361]
	mouse model of heteroplasmic PMD	pre-clinical	[366,367]
Allotopic gene expression	cybrids with m.8993T>G mutation	pre-clinical	[369]
	rat model of LHON	pre-clinical	[370]
	LHON patients	clinical (NTC01267422; NCT02064569)	[371,372]
Mitochondrial augmentation therapy	children with Pearson syndrome	clinical	Unpublished
	children with KSS or Pearson Syndrome	clinical (NCT03384420)	unpublished
	KSS patient	clinical	[375]
Precision medicine approaches for PMD caused by nuclear defects			
Gene therapy approaches	*Ant1* ko mouse model	pre-clinical	[381]
	*Tymp* ko mouse model	pre-clinical	[390]
	Ethe1 ko mouse model	pre-clinical	[389]
	*Ndufs4* mouse model	pre-clinical	[391]
	*Mpv17* ko mouse model	pre-clinical	[58]
	mouse model of Leigh syndrome	pre-clinical	[382]
Liver transplantation	PMD patients	clinical	[385]
	DGUOK-deficient patients	clinical	[386]
	25-year-old MNGIE patient	clinical	[395]
	ETHE1 patients	clinical	[396,397]
Cell replacement	MNGIE patients	clinical	[400,401]
Molecular bypass therapy in disorders of mtDNA instability	*Tk2* mouse model	pre-clinical	[405,406,407]
	DGUOK mutant fibroblasts	pre-clinical	[404]
	RRM2B mutant fibroblasts	pre-clinical	[408]
	*dguok^−/−^* zebrafish	pre-clinical	[409]
	early-onset TK2-patients	clinical (open-label study)	[410]
